# A stacking ensemble deep learning approach to cancer type classification based on TCGA data

**DOI:** 10.1038/s41598-021-95128-x

**Published:** 2021-08-02

**Authors:** Mohanad Mohammed, Henry Mwambi, Innocent B. Mboya, Murtada K. Elbashir, Bernard Omolo

**Affiliations:** 1 School of Mathematics, Statistics and Computer Science, University of KwaZulu-Natal, Pietermaritzburg, Private Bag X01, Scottsville, 3209 South Africa; 2 Division of Mathematics and Computer Science, University of South Carolina-Upstate, 800 University Way, Spartanburg, USA; 3 School of Public Health, Faculty of Health Sciences, University of Witwatersrand, Johannesburg, South Africa; 4 Department of Epidemiology and Biostatistics, Kilimanjaro Christian Medical University College (KCMUCo), P. O. Box 2240, Moshi, Tanzania; 5 College of Computer and Information Sciences, Jouf University, Sakaka, 72441 Saudi Arabia; 6 Faculty of Mathematical and Computer Sciences, University of Gezira, Wad Madani, 11123 Sudan

**Keywords:** Machine learning, Network models, Computational models, Learning algorithms

## Abstract

Cancer tumor classification based on morphological characteristics alone has been shown to have serious limitations. Breast, lung, colorectal, thyroid, and ovarian are the most commonly diagnosed cancers among women. Precise classification of cancers into their types is considered a vital problem for cancer diagnosis and therapy. In this paper, we proposed a stacking ensemble deep learning model based on one-dimensional convolutional neural network (1D-CNN) to perform a multi-class classification on the five common cancers among women based on RNASeq data. The RNASeq gene expression data was downloaded from Pan-Cancer Atlas using *GDCquery* function of the *TCGAbiolinks* package in the *R* software. We used least absolute shrinkage and selection operator (LASSO) as feature selection method. We compared the results of the new proposed model with and without LASSO with the results of the single 1D-CNN and machine learning methods which include support vector machines with radial basis function, linear, and polynomial kernels; artificial neural networks; k-nearest neighbors; bagging trees. The results show that the proposed model with and without LASSO has a better performance compared to other classifiers. Also, the results show that the machine learning methods (SVM-R, SVM-L, SVM-P, ANN, KNN, and bagging trees) with under-sampling have better performance than with over-sampling techniques. This is supported by the statistical significance test of accuracy where the *p*-values for differences between the SVM-R and SVM-P, SVM-R and ANN, SVM-R and KNN are found to be *p* = 0.003, *p* =  < 0.001, and *p* =  < 0.001, respectively. Also, SVM-L had a significant difference compared to ANN *p* = 0.009. Moreover, SVM-P and ANN, SVM-P and KNN are found to be significantly different with *p*-values *p* =  < 0.001 and *p* =  < 0.001, respectively. In addition, ANN and bagging trees, ANN and KNN were found to be significantly different with *p*-values *p* =  < 0.001 and *p* = 0.004, respectively. Thus, the proposed model can help in the early detection and diagnosis of cancer in women, and hence aid in designing early treatment strategies to improve survival.

Recent global public health research shows an epidemiological paradigm shift from infectious to non-communicable diseases, the latter including different types of cancers. The incidence and prevalence of cancer are on the increase worldwide, both in the developing and developed countries^1,2^. The global cancer statistics estimated about 19.3 million new cancer cases in 2020 alone, and close to 10 million deaths of 36 cancers in 185 countries^3^. Breast cancer (with estimated 2.3 million new cases) is the most common diagnosed among women, followed by lung, colorectal, thyroid, and ovarian cancers. Moreover, the most leading cause of death is the lung cancer (with estimated 1.8 million deaths). The cancer burden is expected to increase to 28.4 million cases by 2040^3^.

Cancer tumor classification based on morphological characteristics alone has serious limitations in differentiating among cancer tumors and may cause a strong bias in identifying the tumor by experts^4–6^. Recently, RNASeq gene expression data^7,8^ has emerged as the preferred technology for the simultaneous quantification of gene expression compared to the DNA microarray^9,10^. The classification of cancer using gene expression data from RNASeq technology provides the opportunity to discriminate healthy and diseased samples or among different types and subtypes of cancer more accurately^11^. RNASeq gene expression data have had a profound impact on disease diagnoses and prognoses through accurate disease classification, which has helped clinicians to choose the appropriate treatment plans for patients^12^. There exists striking disparities in the global cancers among women^3,13^. Correct classification of these cancers is among the essential strategies to inform clinical decisions and reduce morbidity and mortality from cancers among women.

Although the use of gene expression data from RNASeq technology has improved cancer classification, it has its own limitations due to it being characterized by small samples sizes, with each sample having a large number of genes (the curse of dimensionality)^14,15^. In addition, the samples also contain several genes that are uninformative and degrade the classification performance^11,16^. As a way to mitigate this problem, it has been suggested to first perform filtration and feature selection through methods such as the two-sample *t*-test at a given stringent significance threshold before going further with model building^17^. This procedure ensures that only informative and sufficiently differentially expressed genes between the outcome classes are used in building the classifiers. This process of feature selection motivates the evaluation of methods for the classification of different cancer tumors and disease stages, to improve early detection and the design of targeted treatment strategies that may reduce mortality. The two-sample *t*-test as a method for feature selection is easy to use but comes with the problem of multiple testing that the user has to deal with. Other methods or approaches that are model based, such as regularized regression methods, have recently become popularly used.

There are many supervised and unsupervised machine learning as well as deep learning methods developed for cancer classification using gene expression data. Several studies reported a higher predictive performance of the machine learning methods on the multi-class cancer classification problem^11,18–20^. These studies, however, differ in the methods used for feature (gene) selection. In particular, Castillo et al.^18^ used differential expression analysis and minimum-redundancy maximum-relevance method for feature selection in the microarray and RNASeq data. García-Díaz et al.^11^ applied a grouping genetic algorithm for feature selection in five different cancers using RNASeq data.

Ramaswamy et al.^19^, on the other hand, used support vector machines (SVM) and a recursive feature elimination method to remove the uninformative genes. These studies concentrated on the application of machine learning methods on a multi-class classification problem. Several methods developed by other authors for multi-class cancer classification are reported to have a higher predictive performance compared to existing methods^21^. Lee et al*.*^22^ proposed a new ensemble classifier called cancer predictor using an ensemble model (CPEM), for classification of over 31 different cancer tumors downloaded from TCGA repository. In addition, they assessed different input features such as mutation profiles, mutations rates, mutation spectra, and signature. Thereafter, they investigated different machine learning and feature selection models in order to find the best model which achieved 84% of accuracy using 10 folds cross-validation. Furthermore, they used the six most common cancers out of 31 types and the model achieved 94% of classification accuracy. However, some of the statistical methods achieved results that are better than machine learning algorithms.

Tabares-Soto et al.^23^ compared machine learning and deep learning methods in classifying 11 different tumor classes using microarray gene expression data. They implemented eight supervised machine learning methods including KNN, support vector classifier (SVC), logistics regression (LR), linear discriminant analysis (LDA), naïve Bayesian classifier (NB), multi-layer perceptron (MLP), decision trees, and random forest (RF) as well as one unsupervised method such as k-means. In addition, they applied two deep neuronal networks (DNN) methods. Their results showed that the deep learning methods outperformed the other machine learning methods.

In this study, we propose a stacking ensemble deep learning model that uses five 1D-CNN as base models. The results of these models are combined using NN, which is used as a meta model to classify the most common types of cancers among women using RNASeq data. We compared the performance of our new proposed model when using the full list of genes as input with its performance when using a reduced selection of genes using LASSO. Also, we consider comparing the performance of our current proposed model with other machine learning methods since there are limited studies that compare the performance of deep learning and machine learning methods to classify different types of cancer. LASSO is used as a feature selection technique, since it has been shown to improve prediction accuracy, especially when there is a small number of observations and a large number of features^24^. Findings from this study might help in the early detection and accurate classification of these cancer types and contribute to efforts of finding therapies that may increase survival for women at risk.

## Material and methods

In this paper, we downloaded the RNASeq gene expression data from Pan-Cancer Atlas (https://portal.gdc.cancer.gov/), using *R* statistical software version 3.6.3 via the *TCGAbiolinks* package^25–27^. The data contains 2166 samples from the top five common cancers between women. We applied eight multi-class classification methods to find the best classifier that discriminates among five common cancers among women. The machine learning methods were implemented in the *R* software, while the deep learning method (1D-CNN) was implemented using *TensorFlow* with *Keras*.

### Datasets

We used only five cancer tumors (normal cases were excluded) from RNASeq gene expression datasets. The cancer tumors were breast, colon adenocarcinoma, ovarian, lung adenocarcinoma, and thyroid cancer. The datasets were downloaded from Pan-Cancer Atlas using *GDCquery* function of the *TCGAbiolinks* package in *R*^26^. *GDCquery* function has many parameters, to define the function known by the following names: project, legacy, data.category, data.type, platform, file.type, experimental.strategy, and sample.type. The project parameter indicates a list of the data that should be downloaded. In our case, we passed the five project codes corresponding to our five types of cancer, which are TCGA-BRCA, TCGA-COAD, TCGA-OV, TCGA-LUAD, and TCGA-THCA. We set the legacy to “true”, which helps the query to search only in the legacy repository for the unmodified stored data in the TCGA data portal.

“Gene expression” and “Gene expression quantification” are passed to the data.category and data.type arguments, respectively, to filter the data files to be downloaded. The platform “Illumina HiSeq” was used to download the data. We used “results” for file.type argument to filter the legacy database, and “RNA-Seq” was chosen as experimental.strategy argument to produce the expression profiles. Moreover, we selected the tumor samples to be downloaded using the “Primary solid Tumor” value as sample.type argument. The downloaded data in a matrix form included five types of cancer, where the columns represent the samples and the rows containing the genes, i.e. features (equivalently covariates). The datasets were combined to give 2166 tumor samples obtained from all the five cancers, with 19,947 common genes. Due to the curse of high dimensionality, we performed filtration and feature selection to reduce the high number of genes in order to exclude irrelevant and noisy ones that could affect the performance of the methods. Thus, we applied normalization, transformation, and filtration steps to the data to select the informative genes that potentially could contribute positively to the classification accuracy. Table 1 below shows a summary of the downloaded data including the training and testing fractions for each cancer tumor.

**Table 1:** Number of samples in each class used in the classification.

Cancer tumor	Number of samples (%)	Training ($\approx$ 70%)	Testing ($\approx$ 30%)
Breast (BRCA)	1082 (50)	753	329
Colon adenocarcinoma (COAD)	135 (6)	99	36
Lung adenocarcinoma (LUAD)	275 (13)	189	86
Ovarian (OV)	304 (14)	217	87
Thyroid (THCA)	370 (17)	259	111
Total	2166	1517	649

### Data pre-processing

**Figure 1:**
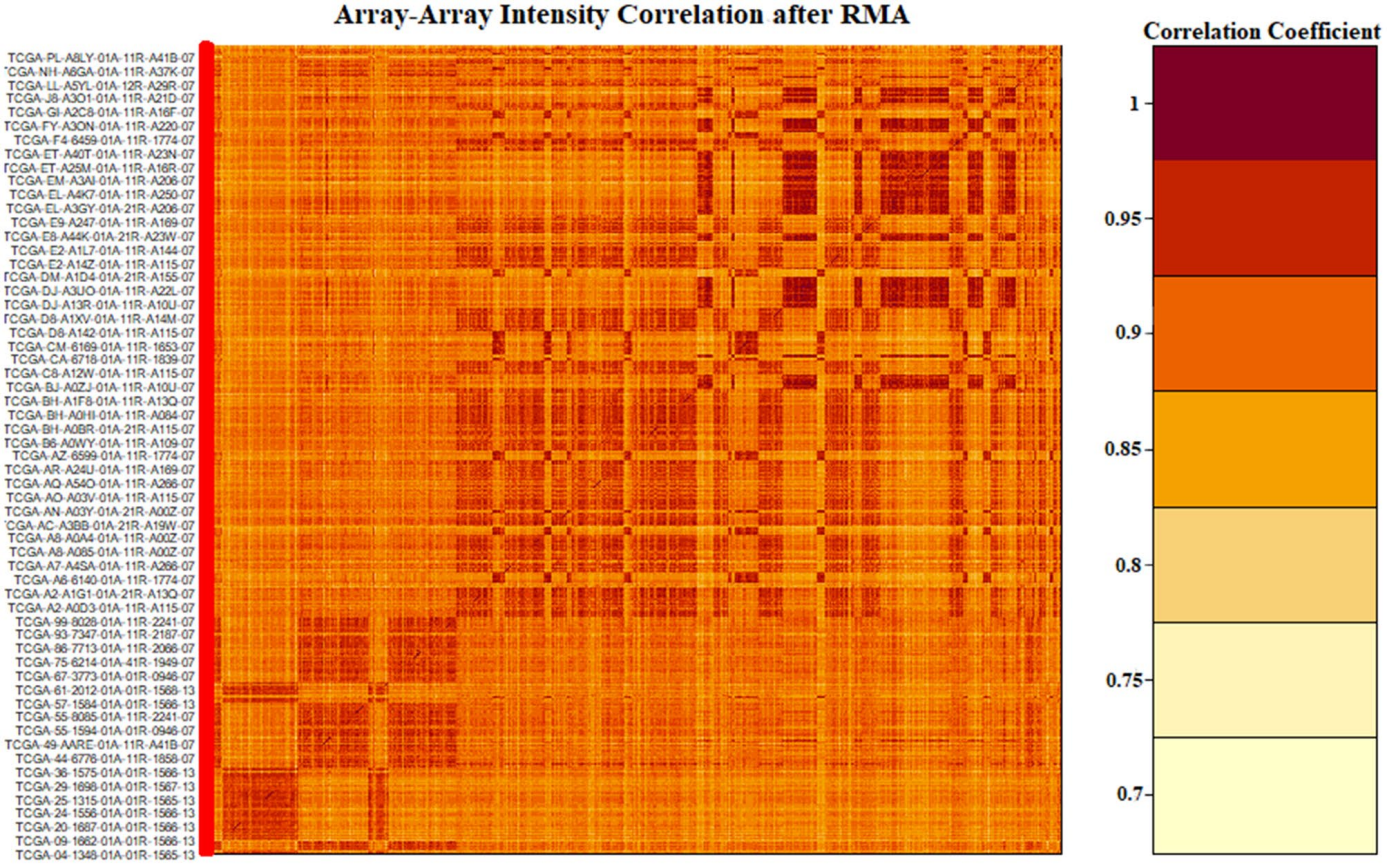
Array-array intensity correlation (AAIC) matrix defines the Pearson correlation coefficients among the samples.

We used *TCGAanalyze_Preprocessing* function in *TCGAbiolinks* package^26^, which utilizes an array-array intensity correlation (AAIC) approach to obtain a $N$ × $N$ square symmetric matrix of Spearman correlations among the samples. The AAIC enabled us to find samples with low correlation considered as possible outliers (Fig. 1). After that, we performed gene normalization through TCGAanalyze_Normalization function, which calls the sub-routines newSeqExpressionSet, withinLaneNormalization, betweenLaneNormalization, and counts from *EDASeq* package to adjust the GC-content effect or other gene level effects, distributional differences between lanes, and global-scaling and full-quantile normalization^28^. *TCGAanalyze_Filtering* was used for filtering out the irrelevant genes and returned the genes with the mean intensity across the samples higher than 0.25, which was the threshold defined quantile mean. After applying this process, we found 14,899 genes to be informative meaning 5048 genes were rendered irrelevant. For further reduction and precise differential gene expression analysis, we used *DESeq* package in *R*^29–31^. *DESeq* analyses the gene expression based on the negative binomial distribution and a shrinkage estimator for the distribution’s variance. After using *DESeq* package, 12,649 genes out of the 14,899 post initial filtering were found to be differentially expressed meaning a further 2250 genes were removed.

### Feature selection using LASSO regression

The RNASeq gene expression data after preprocessing had 12,649 dimensions or features, which was still huge given that the number of samples was 2166. Therefore, LASSO regression was used to decrease the number of genes or features that enabled us to effectively analyze the data. LASSO is a method that performs regularization and feature selection through a shrinkage (regularization) process. LASSO penalizes the regression coefficients with $L1$-norm whereby some coefficients are shrunk to zero. After that, the coefficients of the regression variables having significantly non-zero values are selected and used in the model^24^.

In the case of the multinomial response with $K > 2$ levels, assume that $p_{\ell } \left( {g_{i} } \right) = \Pr \left( {C = c_{i} {|}g_{i} } \right)$, where $c_{i} \in \left\{ {1, 2, 3, \ldots , K} \right\}$ is the *i*th response. The log-likelihood of the multinomial model under LASSO model can be written in a generalized form as^32^

1 $$\begin{array}{*{20}c} {max} \\ {\left\{ {\beta_{0\ell } , \beta_{\ell } } \right\}ss_{1}^{K} \in {\mathbb{R}}^{{K\left( {p + 1} \right)}} } \\ \end{array} \left[ {\frac{1}{N}\mathop \sum \limits_{i = 1}^{N} \log p_{{c_{i} }} \left( {g_{i} } \right) - \lambda \mathop \sum \limits_{\ell = 1}^{K} P_{\alpha } \left( {\beta_{\ell } } \right)} \right].$$

which can be maximized as a penalized log-likelihood.

The outcomes in the data can be denoted in the form of a matrix Y of dimension $N\times K$, with elements $y_{i\ell } = I\left( {c_{i} = \ell } \right)$. Thus, the terms in the regularized log-likelihood in Eq. (1) can be written in more explicit form

2 $$\ell \left( {\left\{ {\beta_{0\ell } , \beta_{\ell } } \right\}_{1}^{K} } \right) = \frac{1}{N}\mathop \sum \limits_{i = 1}^{N} \left[ {\mathop \sum \limits_{\ell = 1}^{K} y_{i\ell } \left( {\beta_{0\ell } + g_{i}^{T} \beta_{\ell } } \right) - \log \left( {\mathop \sum \limits_{\ell = 1}^{K} e^{{\beta_{0\ell } + g_{i}^{T} \beta_{\ell } }} } \right)} \right].$$

3 $$P_{\alpha } \left( \beta \right) = \left( {1 - \alpha } \right)\frac{1}{2}\|\beta\|_{{L_{2} }}^{2} + \alpha \|\beta\|_{{L_{1} }} ,$$

4 $$= \mathop \sum \limits_{j = 1}^{p} \left[ {\frac{1}{2}\left( {1 - \alpha } \right)\beta_{j}^{2} + \alpha \left| {\beta_{j} } \right|} \right].$$

$P_{\alpha }$ is the penalty part, where $g_{i}$ is the gene expression levels for sample $i$, $\beta_{\ell }$ is the vector of the regression coefficients, $y_{i\ell }$ is the class response value in sample $i$. When $\alpha = 0$ in Eq. (3) we obtain the ridge regression penalty, whereas $\alpha = 1$ leads to LASSO regression penalty.

We chose LASSO regression because it uses the sum of the absolute values of the model parameters, restricted to be less than a fixed value as the penalty. LASSO, with tenfold cross-validation returned 173 genes (Supplementary File 1). These genes were obtained when lambda (λ) value gave a minimal deviance associated with the response variable, and so were used for the classification. The cross-validated multinomial deviance is a function of log(λ), and when log(λ) is equal to  − 1, it is an indication that λ and multinomial deviance are both big. As λ decreases and becomes very small, the multinomial deviance also becomes small and almost flat, indicating that the attained model is a good fit.

There are many advantages of the LASSO method, which include removing those variables with zero coefficients that lead to reduced variance without an intrinsic increase in bias. The method also minimizes over-fitting by excluding irrelevant variables that are not related to the outcome variable. The LASSO method naturally also deals with the multiple testing problem, by penalizing irrelevant features, whose contribution is shrunk to zero. This leads to an improved classification and prediction accuracy^24,33^. In our case, LASSO was implemented using *glmnet package* in *R*^34^.

### Data partitioning

We used tenfold cross-validation to evaluate the different prediction methods using 70% of the dataset. In the tenfold cross-validation, the dataset is divided into ten parts, where one part is removed to represent the validation set, and the remaining nine parts combined to represent the training set. Thus, this process is repeated ten times by removing one part each time to have a different part of the data for validation^35^. We left aside 30% of the entire dataset, which served as an independent testing set for the final evaluation.

### The classification models

We performed classification on the different cancers as a multi-classification problem using gene expression levels as covariates. Eight classification methods were used: the new proposed stacking ensemble deep learning model; one-dimensional convolutional neural network (1D-CNN); support vector machines (SVM) with radial basis function, linear, and polynomial kernels; artificial neural networks (ANN); K-nearest neighbors (kNN); and bagging trees.

Support vector machines (SVM)^36^, is a well-known machine learning method that has been used widely in many fields, including gene expression data analysis^37,38^. SVM aims to find an optimal hyperplane that separates the data into two different classes for the binary classification problem, determined by a subset of samples known as support vectors^39^. SVM can handle non-linearly separable problems by transforming the data using mapping kernel functions. These functions include radial basis, polynomial, and linear functions^40^. The SVM is implemented using *kernlab* package in *R* statistical software^41^.

Suppose we have $n$ samples and $p$ genes. Furthermore, assume samples belong to two linearly separable classes represented by + 1 or − 1, and suppose ${\varvec{g}}_{i}$ is the features vector. Then we let, $\left( {{\varvec{g}}_{i} , {\varvec{y}}_{i} } \right) \in G \times Y, i = 1,2,3, \ldots , n$, where $y_{i} \in \left\{ { + 1, - 1} \right\}$ is the target variable dichotomy in the $p$ dimensional space. The aim is to classify the sample into one of the two classes and by extension find an SVM classifier that generalizes to a multi-class problem. There are many hyperplanes that discriminate the two classes, but the goal is achieved by finding an optimal separating hyperplane that lies furthest from the both classes.

The separating hyperplane can be defined by

5 $$\user2{w*g} + b = 0.$$

where ${\varvec{w}}$ is the weight vector, $b$ is the bias, and $\left| b \right|/\left\| {\varvec{w}} \right\|$ is the perpendicular distance to the hyperplane. We can rescale the ${\varvec{w}}$ and $b$ such that the following equation determines the point in each class that is nearest to the hyperplane defined by the equation

6 $$\left| {\user2{w*g} + b} \right| = 1.$$

Therefore, a separating hyperplane for the two classes should follow

7 $$\user2{w*g} + b \ge + 1, \quad when\, y_{i} = + 1.$$

8 $$\user2{w*g} + b \le - 1, \quad when\, y_{i} = - 1.$$

After the rescaling, the distance from the nearest point in each class to the hyperplane becomes $1/\left\| {\varvec{w}} \right\|$. Consequently, the distance between the two classes is $2/\left\| {\varvec{w}} \right\|$, which is called the margin. The solution of the following optimization problem is obtained by maximizing the margin:

9 $$\begin{aligned} & {}_{w, b}^{min} \left\| {\varvec{w}} \right\|^{2} \\ & {\text{subject to}}\quad y_{i} \left( {w*g + b} \right) \ge 1, \quad i = 1,2,3, \ldots , n. \\ \end{aligned}$$

For the multi-class problem there are many types of extensions that can be used such as one-vs-one, one-vs-all (one-vs-rest), decision directed acyclic graph based approach, multi-class objective function, and error-correcting output code based approach. These approaches use the same binary classification principle, where the multi-class problem is decomposed into multiple binary problems. In the one-vs-one multi-class classification problem the SVM classifier produces all possible pairs of binary classifications. Suppose we have $k$ classes where $k > 2$, then, $\frac{{k\left( {k - 1} \right)}}{2}$ binary classifiers are produced in the training step of the algorithm. Consequently, a sample in the test dataset is assigned the class label that is voted the most by the $$\left( {\begin{array}{*{20}c} k \\ 2 \\ \end{array} } \right)$$ binary classifiers from the trained one-vs-one SVM. In our case we use the one-vs-one multi-class classifier.

Artificial neural networks (ANN) is a computational method constructed from many layers, each layer consisting of nodes called neurons^42^. The data flows from the input layer to the output layer through the hidden layers^43^. The nodes between the input through the hidden layers to the output layers are connected by appropriately defined weights or weight functions. The number of input and output layers depends on the number of covariates in the dataset as well as a number of classes in the outcome variable^43^. The inputs are weighted by multiplying every one of them by a weight which is a measure of its contribution. Therefore, the hidden layer receives the weighted inputs and produce outputs using an activation function(s)^40,42^. ANN can be implemented using the *R* package *nnet*^44^.

Specifically suppose we have gene expression data with $p$ genes. The input layer receives the $p$ genes and multiplies them by weights as follow

10 $$b_{i} = \mathop \sum \limits_{j = 0}^{p} w_{ij}^{\left( 1 \right)} g_{i} \quad i = 1,2,3, \ldots , n,$$

where $g$ is a vector of input features and $g_{0} = 1$ is a constant input feature with weight $w_{i0}$. The $b_{i}$ are called activations, and the parameters $w_{ij}^{\left( 1 \right)}$ are the weights. The subscripts (1) refer to the first layer of the network. Then the activations are transformed by a nonlinear activation function $f$, usually a sigmoid function as given in the following equation

11 $$z_{i} = f\left( {b_{i} } \right) = \frac{1}{{1 + {\text{exp}}\left( { - b_{i} } \right)}}.$$

In the second layer, the outputs of the hidden units are linearly combined to give the activations

12 $$h_{k} = \mathop \sum \limits_{i = 0}^{n} w_{ik}^{\left( 2 \right)} z_{i} \quad k = 1,2,3, \ldots , K,$$

where the $w_{ik}^{\left( 2 \right)}$ are the weight parameters for the transformation in the second layer of the neural network. The outputs are transformed using an activation function such as the sigmoid function

13 $$y_{k} = f\left( {h_{k} } \right) = \frac{1}{{1 + {\text{exp}}\left( { - h_{k} } \right)}}.$$

*K*-nearest neighbors (kNN) is a non-parametric method used for classification and regression^45^. The idea behind kNN lies in finding the most nearest neighbors of the new sample, and this is based on the similarity and distance metric^46^. In kNN, *k*-neighbors determine the class of a new instance; therefore, the new sample is assigned the class that is most likely among the *k*-neighbors^40,42^. In general, kNN has two phases; the first is finding the nearest neighbors, and the second is assigning the class of a new sample using those neighbors by the majority vote rule. kNN is implemented using *R* package *caret*^47^.

Suppose we have two samples $s_{1} , s_{2}$ each with $p$ genes. Since kNN uses the Euclidean distance measure to find the closest sample for a new sample, the distance between the two samples can be calculated as

14 $$dist\left( {s_{1} , s_{2} } \right) = \sqrt {\mathop \sum \limits_{j = 1}^{p} \left( {g_{1j} - g_{2j} } \right)^{2} } .$$

A new sample is allocated the class that most of its neighbors fall, that is, model class of its neighbors.

Bagging trees or bootstrap aggregation method is appealing because its ability to reduce the variance associated with a prediction and hence, improve the prediction accuracy^48^. The method splits the data into many bootstrap samples, thereafter, train the model for each bootstrap. Then, the overall prediction obtained by averaging and voting for regression and classification, respectively.

Convolution Neural Networks (CNNs) are deep learning architectures that have multi-layers between the input and output and are designed for image analysis and classification^49–51^. Deep learning is applied successfully in many areas including medical image analysis, computer vision, drug design, and bioinformatics and yield performance that sometimes surpass expert personals' performance^52^. CNNs are a regularized version of fully connected networks (multilayer perceptrons), in which each neuron in one layer is connected to all the neurons in the layer that follows it. The connectivity between the neurons is inspired by the biological process and resembles the arrangement of the animal visual cortex. In contrast to other image classification and analysis algorithms, CNNs use little pre-processing by learning the filters that capture temporal and special dependencies in an image instead of hand-engineering them. A sequence of stacked layers (convolutional layer, pooling layer, and fully-connected layer) makes the architecture of CNNs and in each layer, a differentiable function is used to transform one volume of activations to the layer that follows it. The major building blocks in CNNs are the convolutional layers, which apply filters on an input image to create a feature map. To get a good classification performance, CNNs normally decrease the features of the image into an easier processed arrangement without dropping essential features. The pooling layers use max pooling or average pooling to reduce the dimension of the image’s features. The fully connected layer is an important component in the CNNs architecture that derives the final classification results.

The input to the CNNs is a tensor of order 3 that represents an image having m rows and n columns with 3 color channels (RGB). The tensor encodes the pixel intensities of the image and produces the input features that go through the convolutional, pooling, and the fully connected layers sequentially. In the convolutional layer, a filter of size *f* by *f* and stride = s are applied and the result is 3 × (*m − f* + *1*) × (*n − f* + 1) hidden feature neurons if a stride of 1 is used and the pooling layer result will be 3 × (*m − r* + *1*)*/*2 × (*n − r* + 1)/2 hidden features neurons when applied to 2 × 2 regions. The convolution operation generates the features map by multiplying the element of the input array by the element of the filter element wise and summing up the result to generate on pixel of the features map. Sliding the filter across the matrix and repeating the multiplication and summing up operations will generate the rest of features map pixels. The mathematical equation of this convolution operation is given as follows

15 $$O\left( {i,j} \right) = \mathop \sum \limits_{k = 1}^{f} \left( {\mathop \sum \limits_{l = 1}^{f} input\left( {i + k - 1, j + l - 1} \right)kernel\left( {k,l} \right)} \right)$$

where $$i = 1,2, \ldots m - f + 1$$, $$j = 1,2, \ldots .. n - f + 1$$.

**Figure 2:**
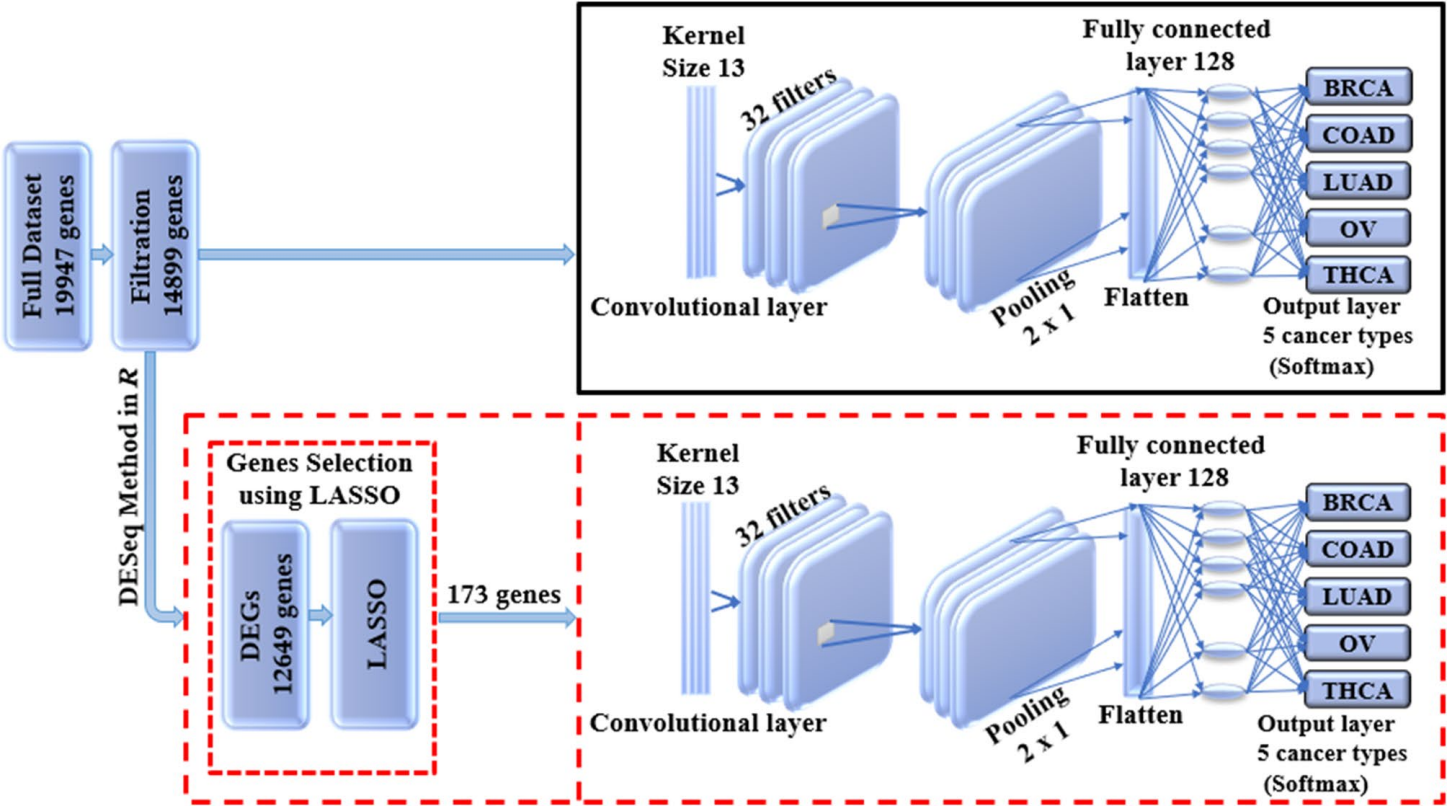
Illustrates the architecture of the 1D-CNN model. The upper panel presents the 1D-CNN without LASSO, while the lower panel shows the usage of LASSO as s feature selection technique for the 1D-CNN where it gives an input vector with 173 genes.

1D-CNN is a simple CNN architecture that has only one convolutional layer. The simple design of this model leads to reduced number of parameters that can be adjusted during the training process therefore, it is highly needed in the genomic studies where it is difficult to collect large data to train a deep learning model that has very large number of parameters^53^. The one dimensional that we used in this study was constructed by Mostavi et al.^53^ for predicting cancer tumor based on gene expression data. The architecture of the model when using LASSO as a feature selection technique is shown in Fig. 2.

### Regularization with early stopping

We applied 1D-CNN with early stopping regularization to avoid over-fitting. The over-fitting is usually caused by training the model too much, making it pick up the noise as an essential part of the data instead of relying only on the training data. Such noise is normally unique to each training data. It can lead to high variance in the model estimates. On the other hand, too little training can result in under-fitting or high bias. Therefore, the variance and the bias have a negative relationship meaning that if the bias increases for fixed mean square error, then the variance will decrease and vice versa and that is known as the bias-variance tradeoff^54,55^. To avoid over-fitting, we can use a model with fewer parameters or obtaining more data. A model with fewer parameters can cause high bias. Since obtaining more data is not easy in the medical field, then a model with fewer parameters seems to be the alternative, but modern approaches in deep learning repeatedly show the benefits of using models with a large number of parameters^56,57^. Therefore, finding a way of adjusting the variance by minimizing noisy data can help solve the over-fitting problem. Since too much training can result in over-fitting, whereas too little training can result in under-fitting then the model can be regularized using the early stopping mechanism. We can implement the early stopping mechanism in the training procedure to make the architectures better fit the training data with each epoch and determining the number of epochs that can be run before the pre-trained model begin to overfit.

### Stacking ensemble

**Figure 3:**
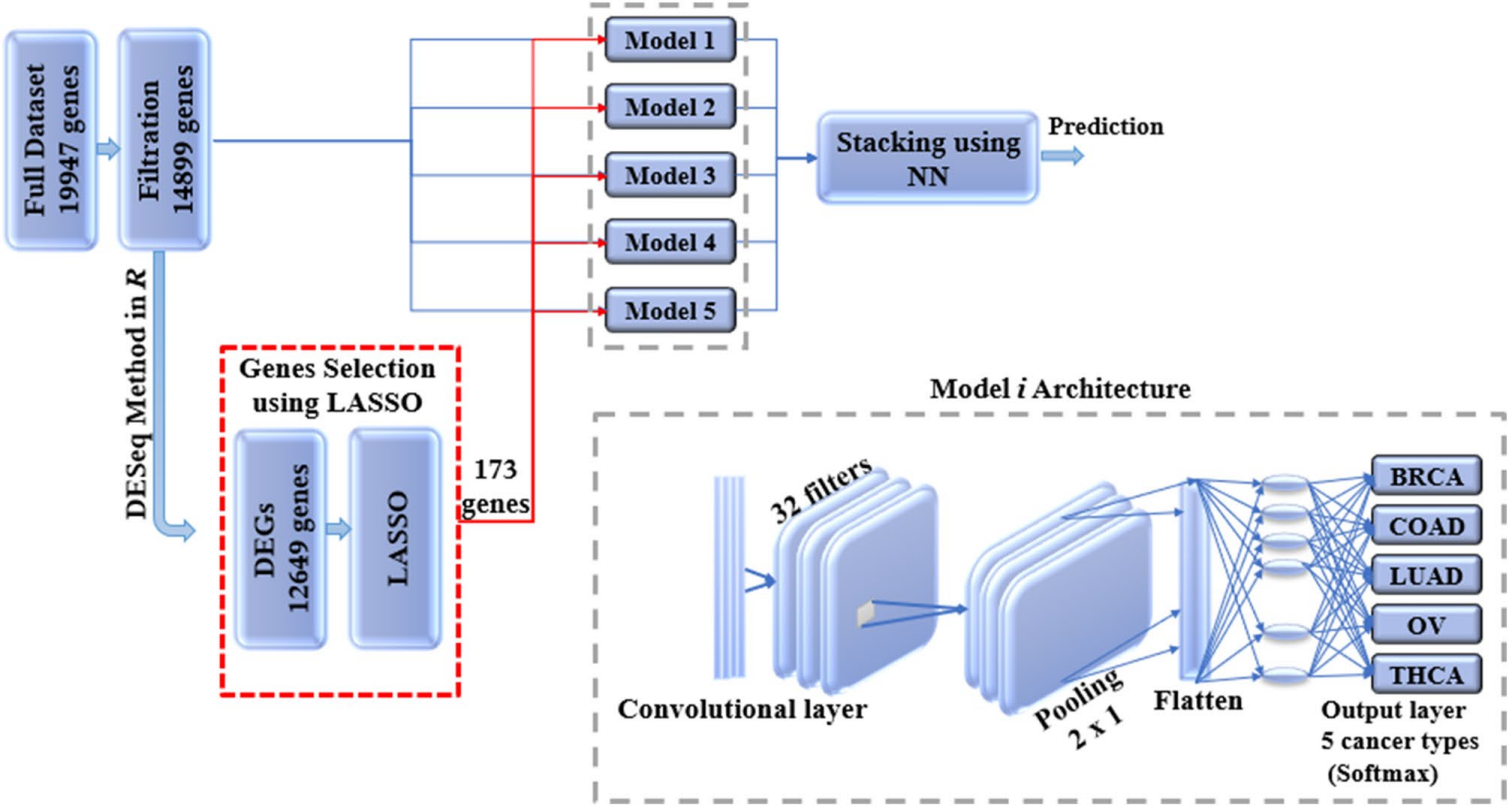
Stacking ensemble deep learning model architecture in which five 1D-CNN models are used as base models and the results of these models are combined using NN, which is used as a meta model. The NN has one hidden layer and an output layer that is activated using softmax function.

Ensemble learning is the process of improving classifiers performance by combining the contribution of the trained sub-models to solve same classification problem^5^. Overall, each base learner votes and the final prediction is gained by the meta-learner, which is a model that learn to correct the prediction of the base-learners. Therefore, the ensemble approach results in prediction accuracy that is better than the single learners. Generalizability of an ensemble usually reduces the variance in the prediction, and thus ensure the most stable and best possible prediction is made. The meta model takes the output of the sub-models (base-learners) as input and then learns to merge the input prediction to make the final prediction which is better than each of the base-classifiers. Figure 3 shows our proposed stacking ensemble deep learning model.

### Performance evaluation

We used different performance metrics to evaluate the performance of the classification methods. These metrics are namely accuracy, kappa, specificity, sensitivity, the area under the curve (AUC), precision, F-measure, and ROC curve. The accuracy measures the percentage of correctly classified cases but is not sufficient for measuring the performance of the classifier, especially if we have unbalanced data (which is the case with cancer data that we are dealing with). Sensitivity measures the percentage of the cases that are correctly classified as having cancer among those samples that are truly cancerous. Therefore, it measures the fraction of the correctly predicted cancer cases. Specificity measures the percentage of cases that do not have cancer, which are correctly identified to be so. In other words, it measures the true negative rate. Precision is the percentage of cases among those classified as positive that are truly positive, i.e., having cancer, and sometimes this measure is called the positive predicted value. F-measure is a measure that balances between precision and sensitivity.

We also compared the predictive performance of the methods using the receiver operating characteristic (ROC) curve plots. These figures were plotted using *MultiROC* package in *R*^58^. *MultiROC* calculates and visualizes ROC curve for multi-class using *micro-averaging* and *macro-averaging* approaches. *Micro-averaging* ROC-AUC converts the multi-class classification into binary classification by stacking all groups together. *Macro-averaging* ROC-AUC uses one versus the rest approach by averaging all group’s results and linear interpolation used between the points of the ROC. Confidence intervals for kappa statistics were computed using *vcd* package.

### Methods to adjust for class imbalances

Imbalanced class sizes may lead to poor predictive performance particularly for the classes with small samples (Table 1). In order to handle the class imbalance and hence improve the models’ performances we used the synthetic minority over-sampling technique (SMOTE) and under-sampling (DOWN) methods. SMOTE has been used widely in various fields such as bioinformatics for addressing the class imbalance in the outcome^59,60^. SMOTE is a data augmentation method that add new data to the minority class that are synthesized from the existing data instead of duplicating the data, because the duplication will not provide any new information to the model. SMOTE works by first selecting randomly a class instance *a* from the minority class then it chooses randomly one of the k nearest neighbors *b* to create the synthetic instances as a convex combination of *a* and *b* and finally, it forms a line segment in the feature space by connecting *a* and *b*.

We synthesized the minority class from existing samples by selecting randomly the closest *k* minority nearest neighbors to balance the class^61–63^. This statistical technique increases and generates the samples to reach the highest majority class and it makes the samples more general. SMOTE is implemented using *caret* package in *R* by adjusting the sampling method in the train control parameter to be ‘SMOTE’.

Under-sampling technique (DOWN) tends to produce a new balanced subset of the original dataset by randomly removing instances usually from the majority class observations^64,65^. DOWN is implemented using *caret* package in *R* by adjusting the sampling method in the train control parameter to be ‘DOWN’.

### Statistical significance test

There are many different techniques that can used for comparing the accuracies of the machine learning models. In this work, we used the *resamples* method in *R* to analyze and visualize the estimated performance of the models. We used the *summary* function to compute summary statistics across each model/metric combination. *Diff* function in *R* is used to estimate the differences between the methods. The *diff* function performs a pairwise comparisons to compute the differences between pairs of consecutive elements using Bonferroni correction as an adjustment method. Bonferroni test is a type of multiple testing method used in statistical analysis to reduce the instance of a false positive and prevent the data from appearing incorrectly to be statistically significant^66,67^.

## Results

**Figure 4:**
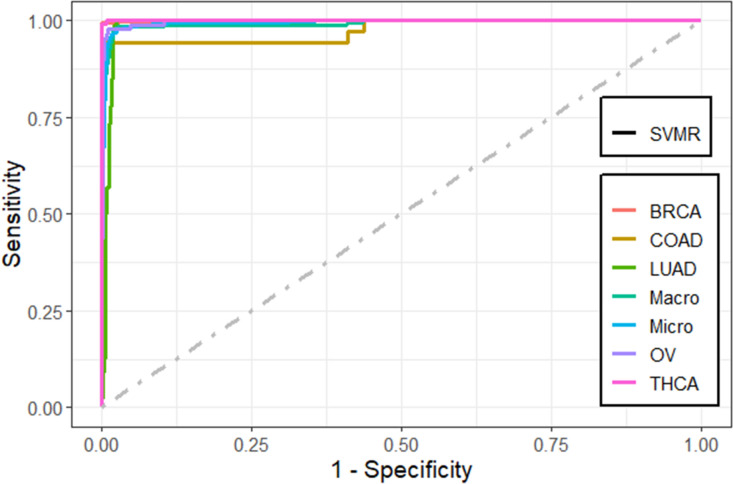
Multi-class ROC curves visualization for the SVMR model based on under-sampling technique.

**Figure 5:**
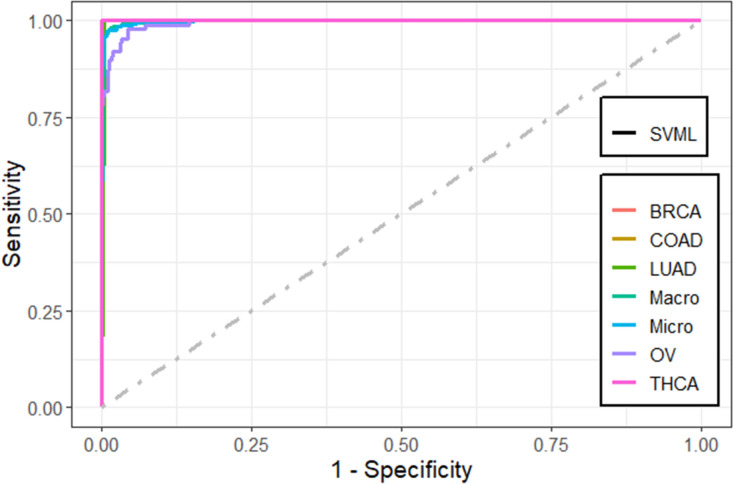
Multi-class ROC curves visualization for the SVML model based on under-sampling technique.

**Figure 6:**
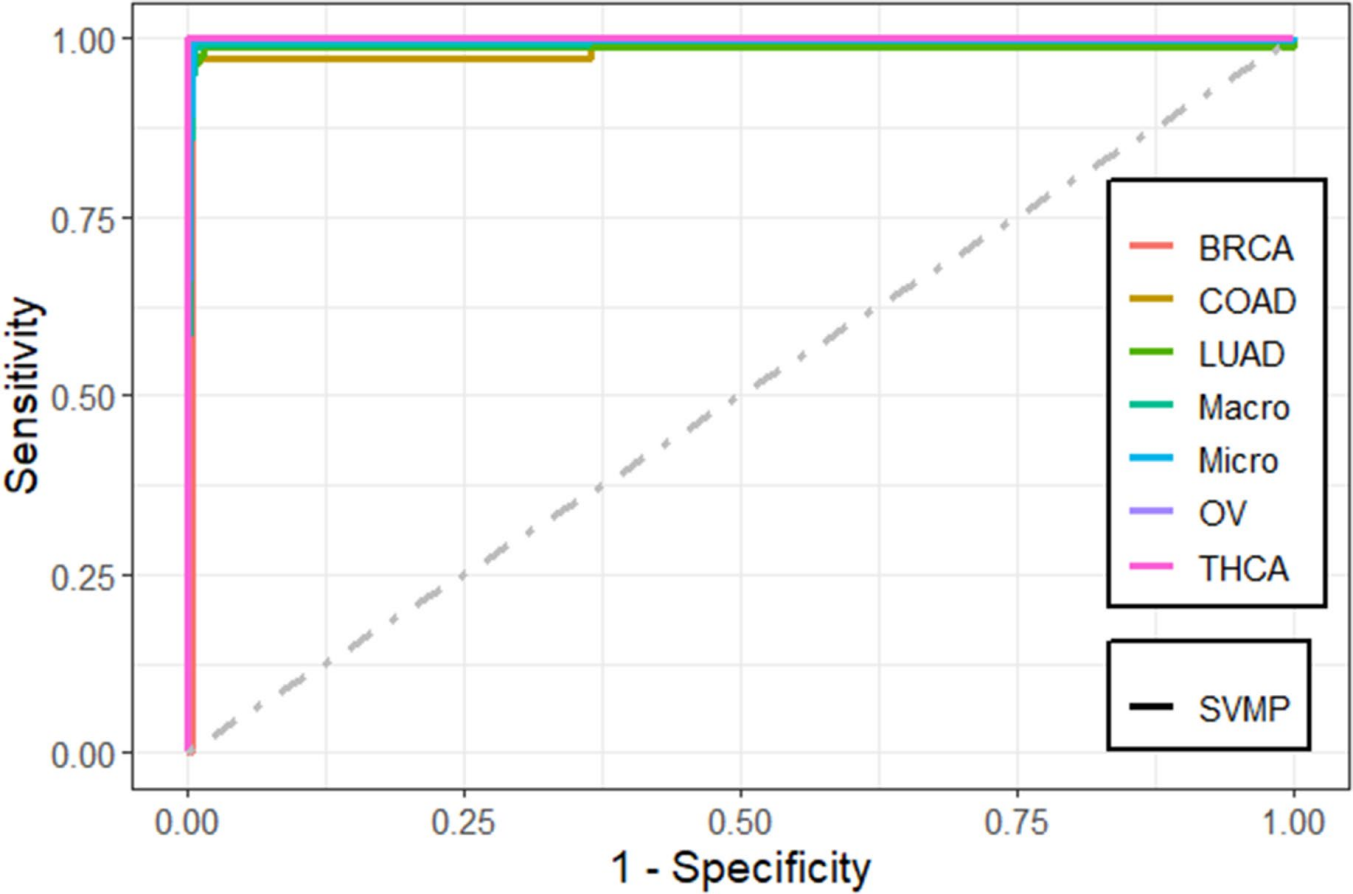
Multi-class ROC curves visualization for the SVMP model based on under-sampling technique.

**Figure 7:**
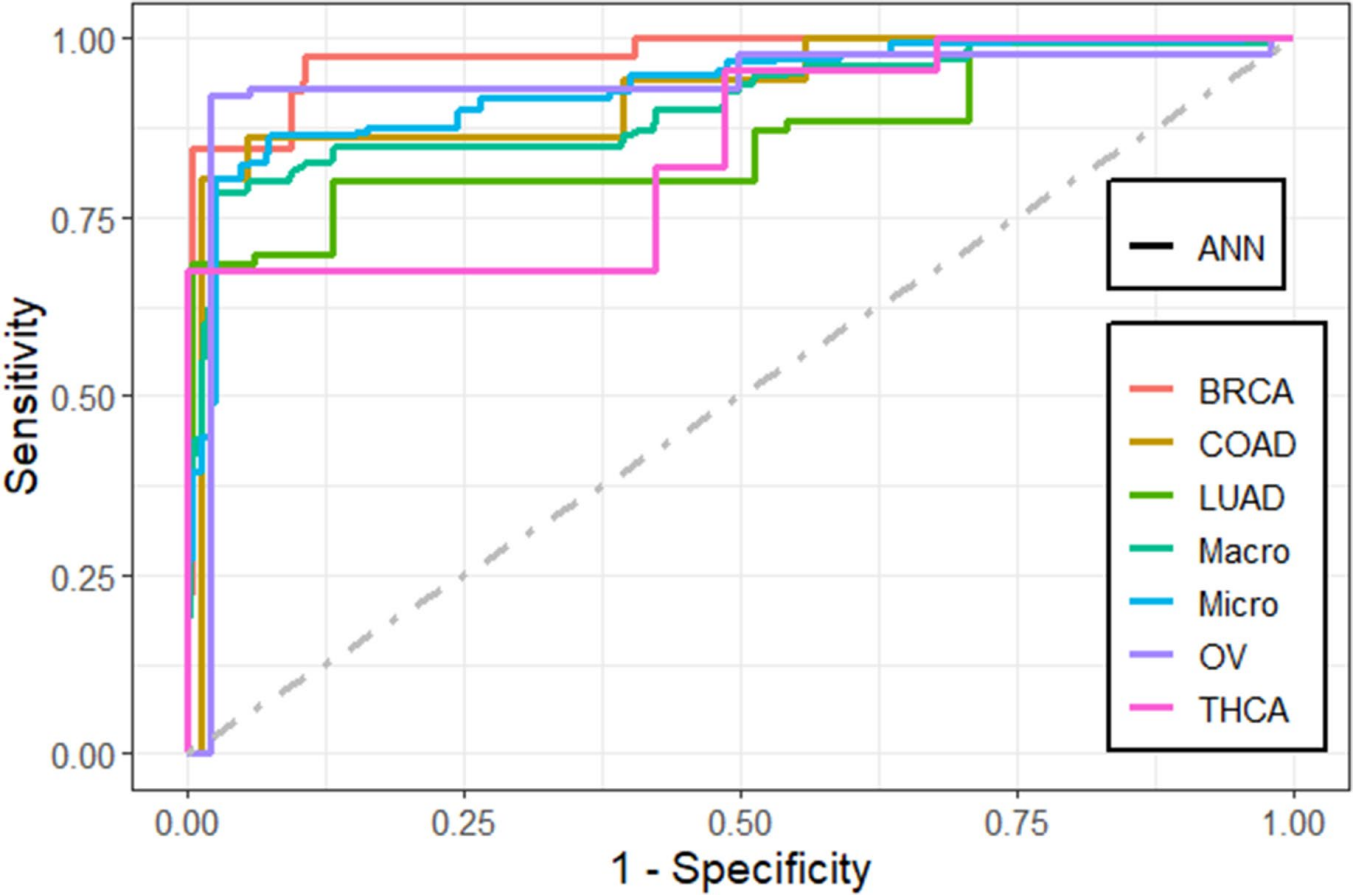
Multi-class ROC curves visualization for the ANN model based on under-sampling technique.

**Figure 8:**
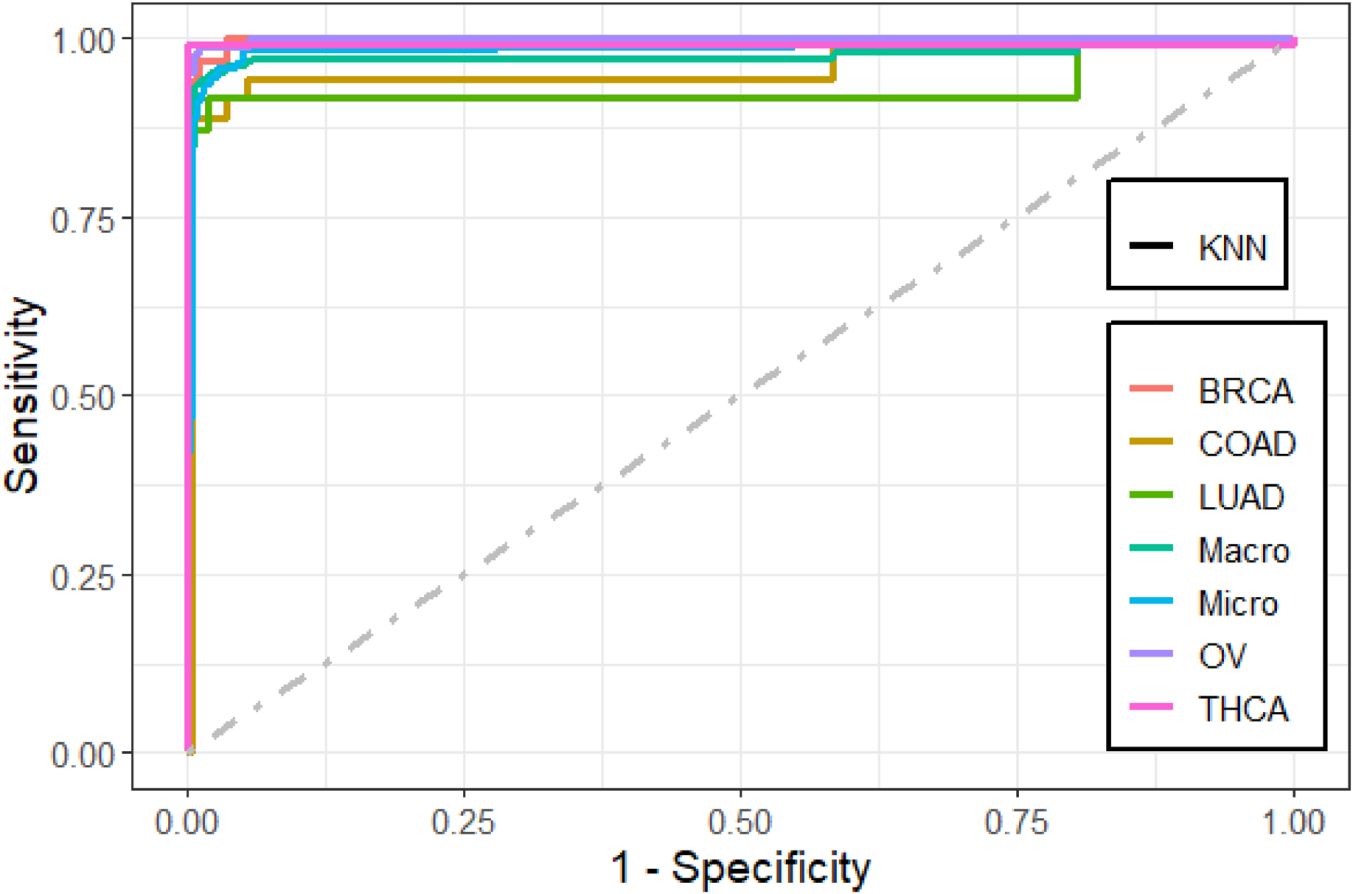
Multi-class ROC curves visualization for the KNN model based on under-sampling technique.

**Figure 9:**
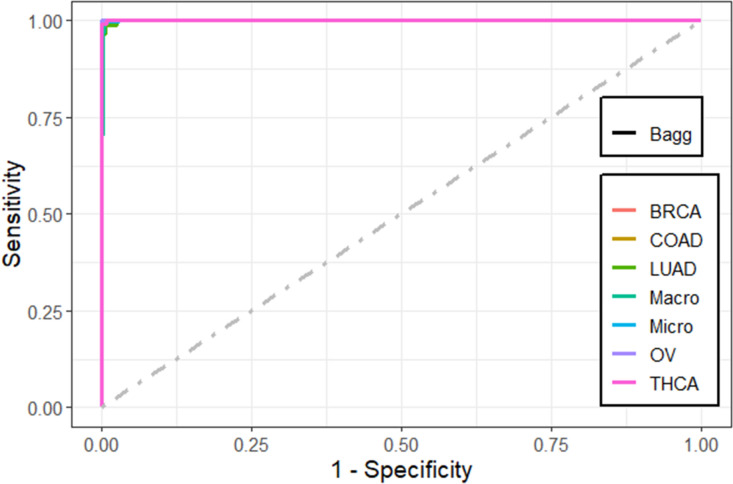
Multi-class ROC curves visualization for the bagging trees model based on under-sampling technique**.**

We found that the performance of the machine learning methods when LASSO as feature selection technique used is by far better than when it is not used. The performance of the methods in terms of overall statistics are summarized in Table 2 based on the under-sampling technique. Table 3 shows the results of methods in terms of per-class statistics for under-sampling technique. The receiver operating characteristic (ROC) curve plots comparing the machine learning classification methods in this study are shown in, Figs. 4, 5, 6, 7, 8 and 9 based on under-sampling method. The predictive performance of the under-sampling technique outperformed the over-sampling technique. Results for the over-sampling technique are available in the Supplementary File 2.

**Table 2:** The overall predictive performance of the machine learning methods based on under-sampling.

Methods	Performance measures					
	ACC (95% CI)	Kappa (95% CI)	F1-Score	Precision	Sensitivity	AUC
SVM-R	95.84 (94.00, 97.24)	93.81 (91.55, 96.07)	98.64	99.39	97.90	98.04
SVM-L	96.76 (95.10, 97.99)	95.14 (92.74, 97.18)	97.48	100.0	95.08	98.56
SVM-P	98.92 (97.79, 99.57)	98.40 (97.89, 99.74)	99.24	99.69	98.79	99.50
ANN	80.74 (77.49, 83.71)	72.15 (70.39, 79.59)	87.46	84.80	90.29	83.84
kNN	93.07 (90.83, 94.90)	89.97 (87.18, 92.75)	95.91	92.70	99.34	94.94
Bagging trees	99.20 (98.21, 99.75)	98.86 (97.86, 99.85)	99.54	99.69	99.39	99.54

*SVM-R* support vector machine with radial-basis function (RBF) kernel, *SVM-L* support vector machine with linear kernel, *SVM-P* support vector machine with polynomial kernel, *ANN* Artificial Neural Networks, *kNN* K-nearest neighbors, Bagging trees; *ACC* accuracy, *CI* confidence interval, *Kappa* kappa statistics, *AUC* area under the curve.

**Table 3:** Predictive performance of the machine learning methods per-class statistics based on under-sampling.

Performance measures	Methods						
	Class	SVM-R	SVM-L	SVM-P	ANN	kNN	Bagging trees
Accuracy	BRCA	98.6	97.3	99.2	87.7	96.0	99.5
	COAD	95.8	98.6	98.6	90.2	94.7	98.5
	LUAD	97.7	99.6	98.0	82.8	90.6	98.7
	OV	90.7	88.5	98.9	93.4	98.5	100
	THCA	97.8	100	100	82.5	99.1	99.6
Sensitivity	BRCA	99.4	100	99.7	84.8	92.7	99.7
	COAD	91.7	97.2	97.2	86.1	94.4	97.2
	LUAD	98.8	100	96.5	68.6	81.4	97.7
	OV	81.6	77.0	97.7	92.0	98.9	100
	THCA	95.5	100	100	67.6	98.2	99.1
Specificity	BRCA	97.8	94.7	98.8	90.6	99.4	99.4
	COAD	100	100	100	94.3	94.9	99.8
	LUAD	96.6	99.3	99.5	97.0	99.8	99.6
	OV	99.8	100	100	94.8	98.0	100
	THCA	100	100	100	97.4	100	100
F1-score	BRCA	98.6	97.5	99.2	87.5	95.9	99.5
	COAD	95.7	98.6	98.6	60.8	67.3	97.2
	LUAD	89.5	97.7	96.5	72.8	89.2	97.7
	OV	89.3	87.0	98.8	81.6	93.5	100
	THCA	97.7	100	100	75.0	99.1	99.6
Precision	BRCA	97.9	95.1	98.8	90.3	99.4	99.4
	COAD	100	100	100	47.0	52.3	97.2
	LUAD	81.7	95.6	96.5	77.6	98.6	97.7
	OV	98.6	100	100	73.4	88.7	100
	THCA	100	100	100	84.3	100	100

*SVM-R* support vector machine with radial-basis function (RBF) kernel, *SVM-L* support vector machine with linear kernel, *SVM-P* support vector machine with polynomial kernel, *ANN* Artificial Neural Networks, *kNN* K-nearest neighbors, Bagging trees, *ACC* Accuracy, *CI* confidence interval, *Kappa* kappa statistics *AUC* area under the curve.

### The overall predictive performance of the machine learning methods based on the under-sampling technique

The accuracy, precision, sensitivity, and F1-Score performance measures for the overall multi-class classification problem based on the under-sampling technique (DOWN) are presented in Table 2. These results show that bagging trees method achieved the best performance measure compared to the other methods where it yields an accuracy, sensitivity, AUC, and F1-score of 99.2%, 99.4%, 99.54, and 99.5%, respectively. However, SVM-P and bagging trees have the same precision, and they have a close results in the other performance measures. Consequently, ANN method obtained the worst performance with an accuracy of 80.7%.

### Predictive performance of the machine learning methods per cancer tumor based on the under-sampling

The accuracy, precision, sensitivity, and F1-Score performance measures based on per-class statistics using the under-sampling technique method (DOWN) are presented in Table 3. Bagging trees outperforms the other methods in classifying most of the five cancer tumors in most of the performance measures, followed by SVM-P method. While the ANN shows the lowest performance measures. These results were confirmed using the ROC curves which are depicted in Figs. 4, 5, 6, 7, 8, and 9. Bagging trees was able to highly correctly classify the ovarian cancer with 100% in terms of accuracy, sensitivity, specificity, F1-Score, and precision. While SVM-L and SVM-P can sensitively classify the thyroid cancer with a 100% of accuracy, sensitivity, specificity, F1-Score, and precision. Also, SVM-R shows performance that is close to SVM-L and SVM-P to classify the thyroid cancer.

### Predictive performance of the one-dimensional convolutional neural network model

The results that are presented in Table 4 show that the 1D-CNN model has a high performance when applied on the genes that are selected using LASSO (173 genes) where it achieved an average classification accuracy of 99.22%. These results also showed that the 1D-CNN outperformed the results of the machine learning methods that are presented in Table 2. It can be noted from the overlapped confusion matrix of the multiclass classification that the deep learning model classified the five categories of the cancers types using the 173 genes better than classifying these categories using the full list of genes (14,899). The resulting precision, recall, and F1-score values are 99.32%, 99.09%, and 99.19%, respectively.

**Table 4:** The performance of the 1D-CNN model using early stopping regularization.

Performance measures	Folds										Overall
	1	2	3	4	5	6	7	8	9	10	
**All (14,899 genes)**											
Accuracy	99.54	98.16	95.85	97.24	97.24	97.24	99.54	96.30	99.54	100	98.06
Precision	99.47	96.07	93.50	96.72	96.92	95.11	99.82	94.16	99.38	100	97.12
Recall	99.26	98.20	96.56	95.22	96.82	96.06	99.26	94.94	99.81	100	97.61
F1-score	99.36	97.03	94.87	95.94	96.78	95.48	99.53	94.54	99.59	100	97.31
**Reduced (173 genes)**											
Accuracy	98.62	99.54	99.08	98.62	99.54	100	99.07	99.54	98.61	99.54	99.22
Precision	99.46	99.31	99.10	98.99	99.82	100	98.48	99.29	98.92	99.82	99.32
Recall	97.97	99.82	99.10	98.39	99.29	100	98.72	99.81	98.52	99.26	99.09
F1-score	98.68	99.56	99.10	98.65	99.54	100	98.57	99.54	98.69	99.53	99.19

**Figure 10:**
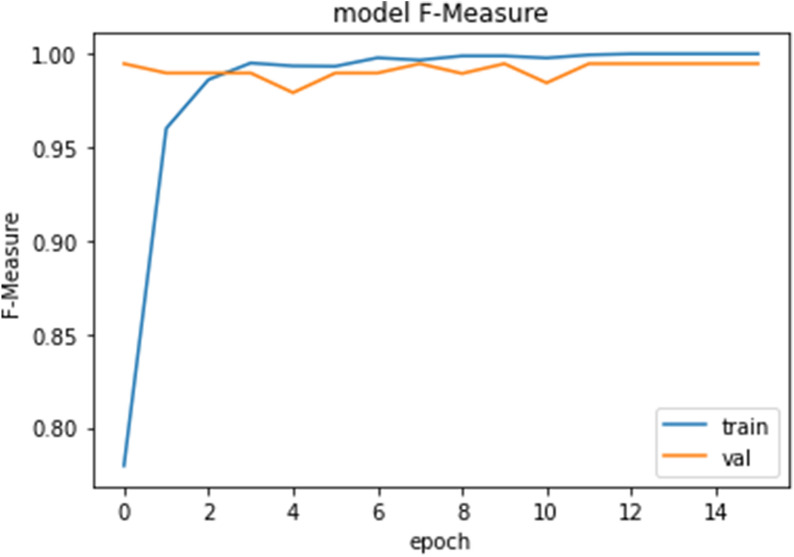
Training and validation F1 measure for the full list of genes with early stopping.

**Figure 11:**
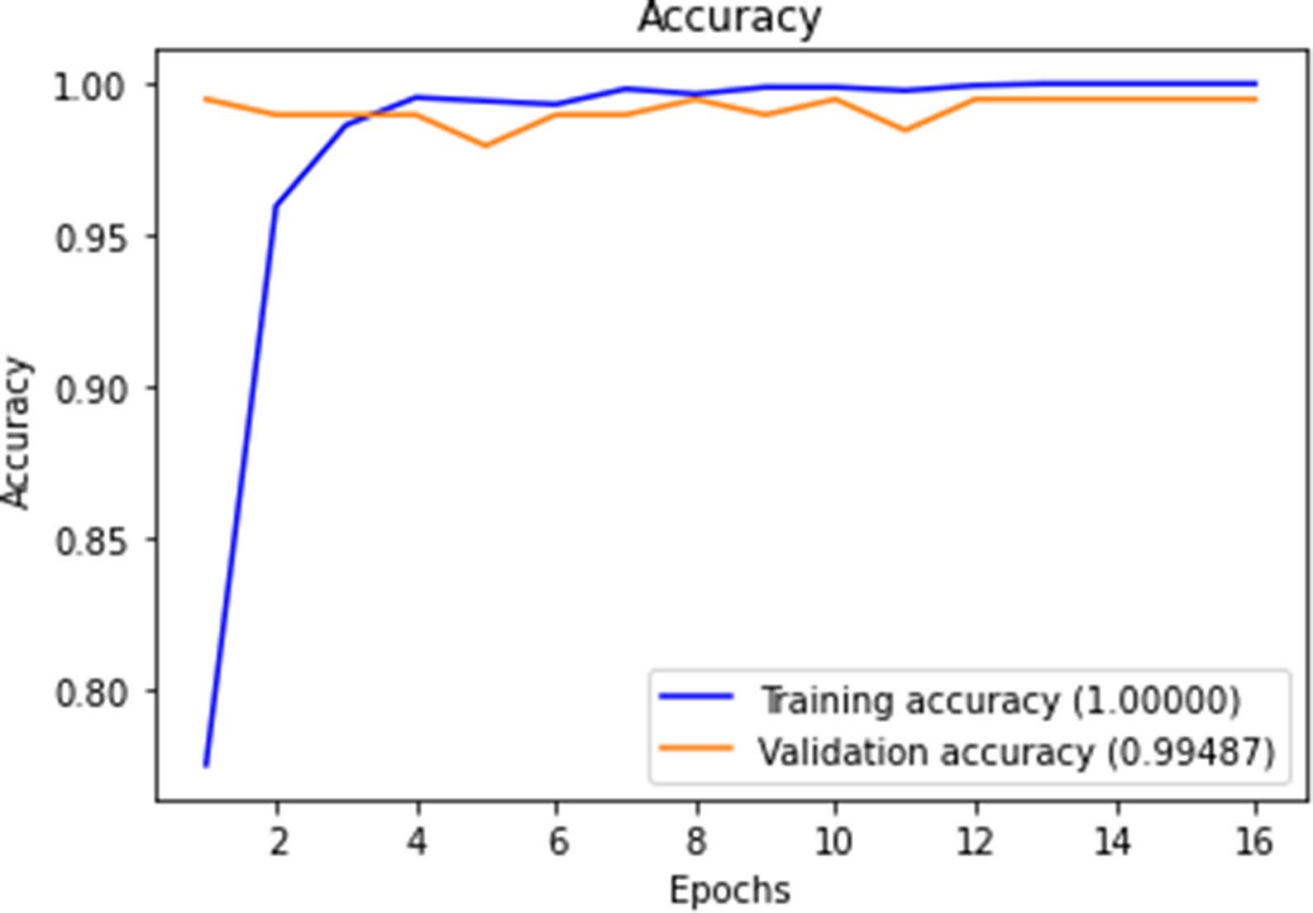
Training and validation accuracy for the full list of genes with early stopping.

**Figure 12:**
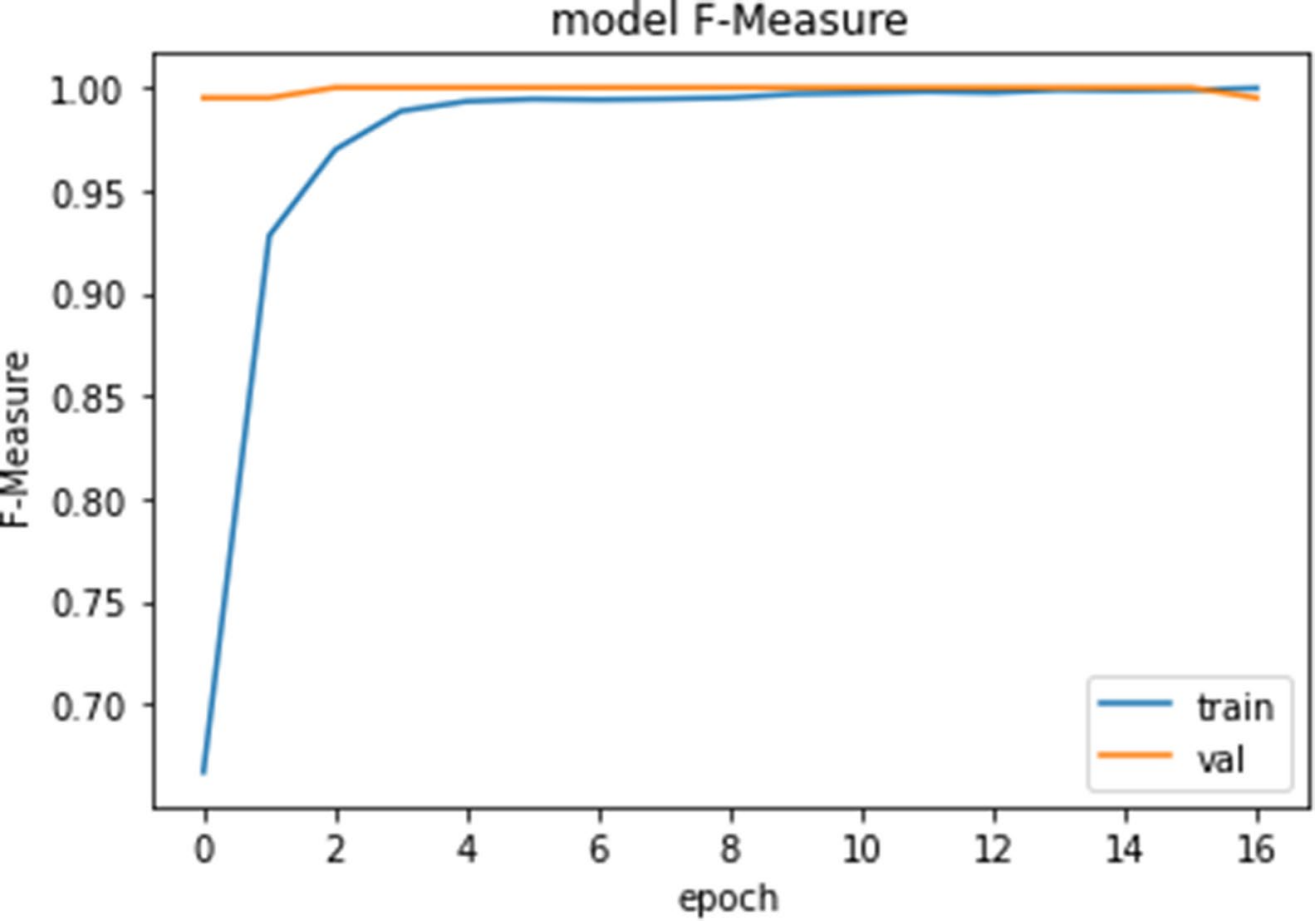
Training and validation F1 measure for reduced genes with early stopping.

**Figure 13:**
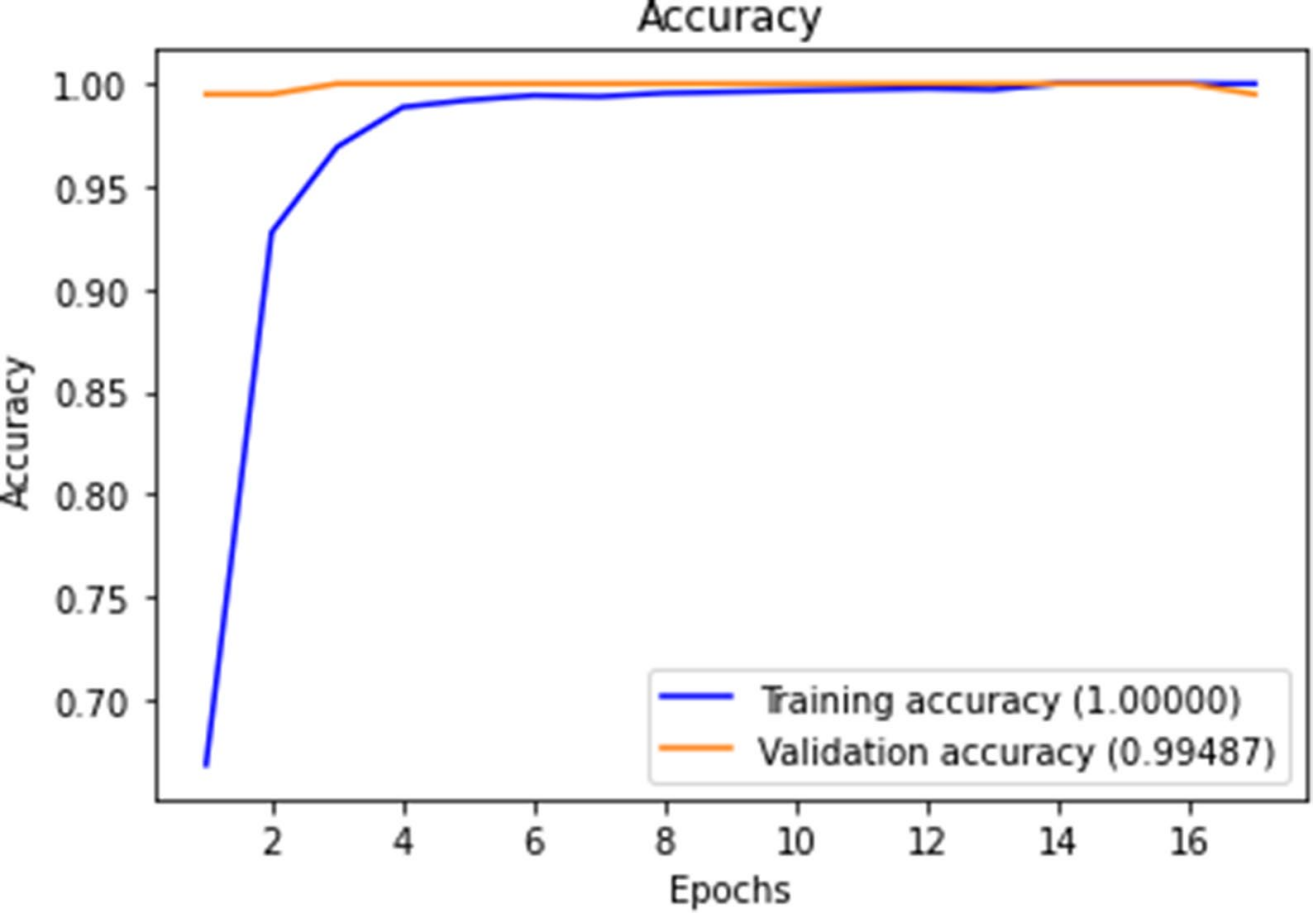
Training and validation accuracy for reduced genes with early stopping.

**Figure 14:**
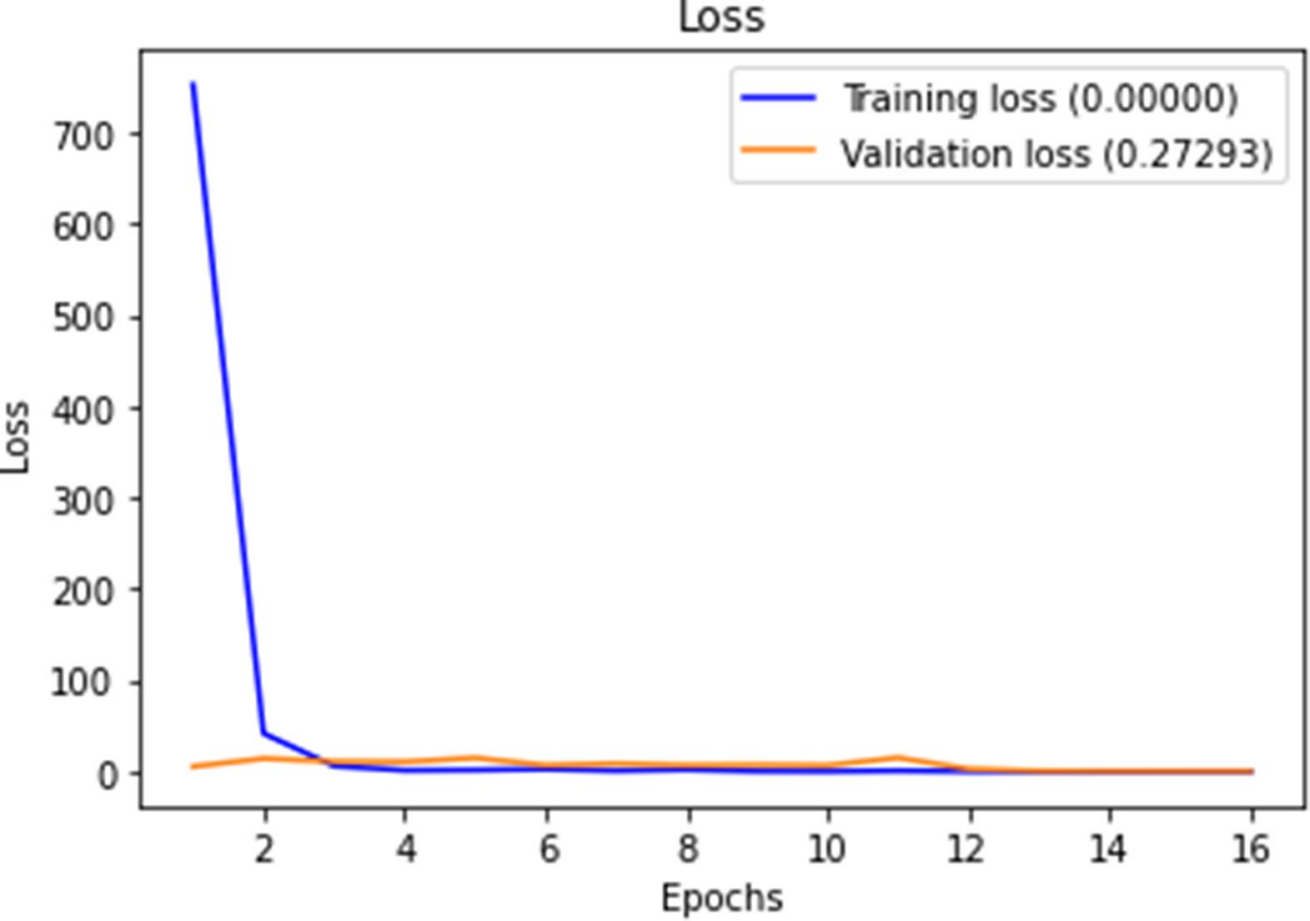
Training and validation loss for the full list of genes with early stopping.

**Figure 15:**
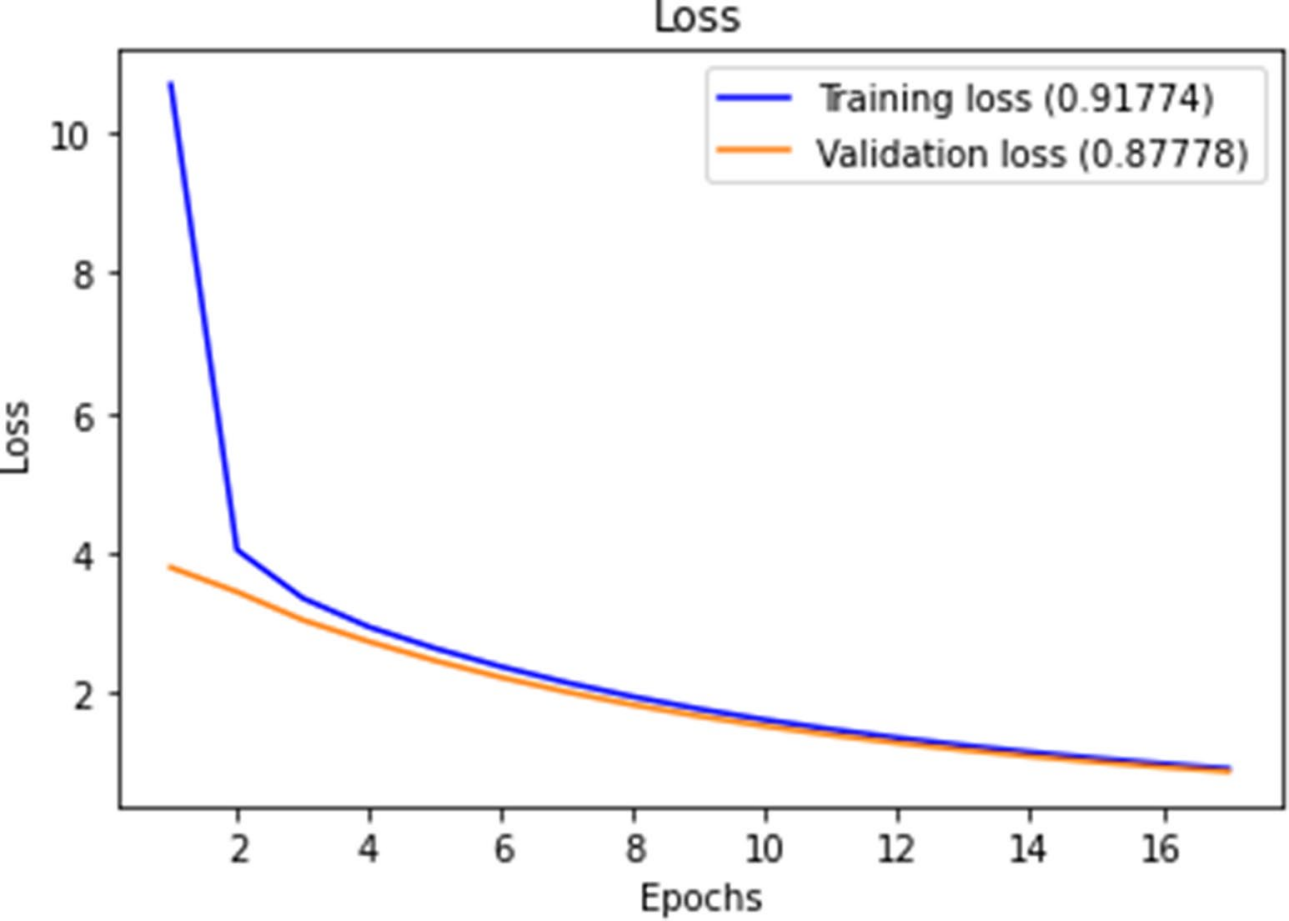
Training and validation loss for reduced genes with early stopping.

Figures 10, 11, 12 and 13 show F1-measure and accuracy for training and validation when training our model using the full list of genes and the reduced genes with the early stopping approach. These figures indicate that the model can generalize very well since they become stable when the F1-measure and the accuracy are more than 99%. Figures 14 and 15 show the losses when using the full list of genes and the LASSO selected genes, respectively.

**Figure 16:**
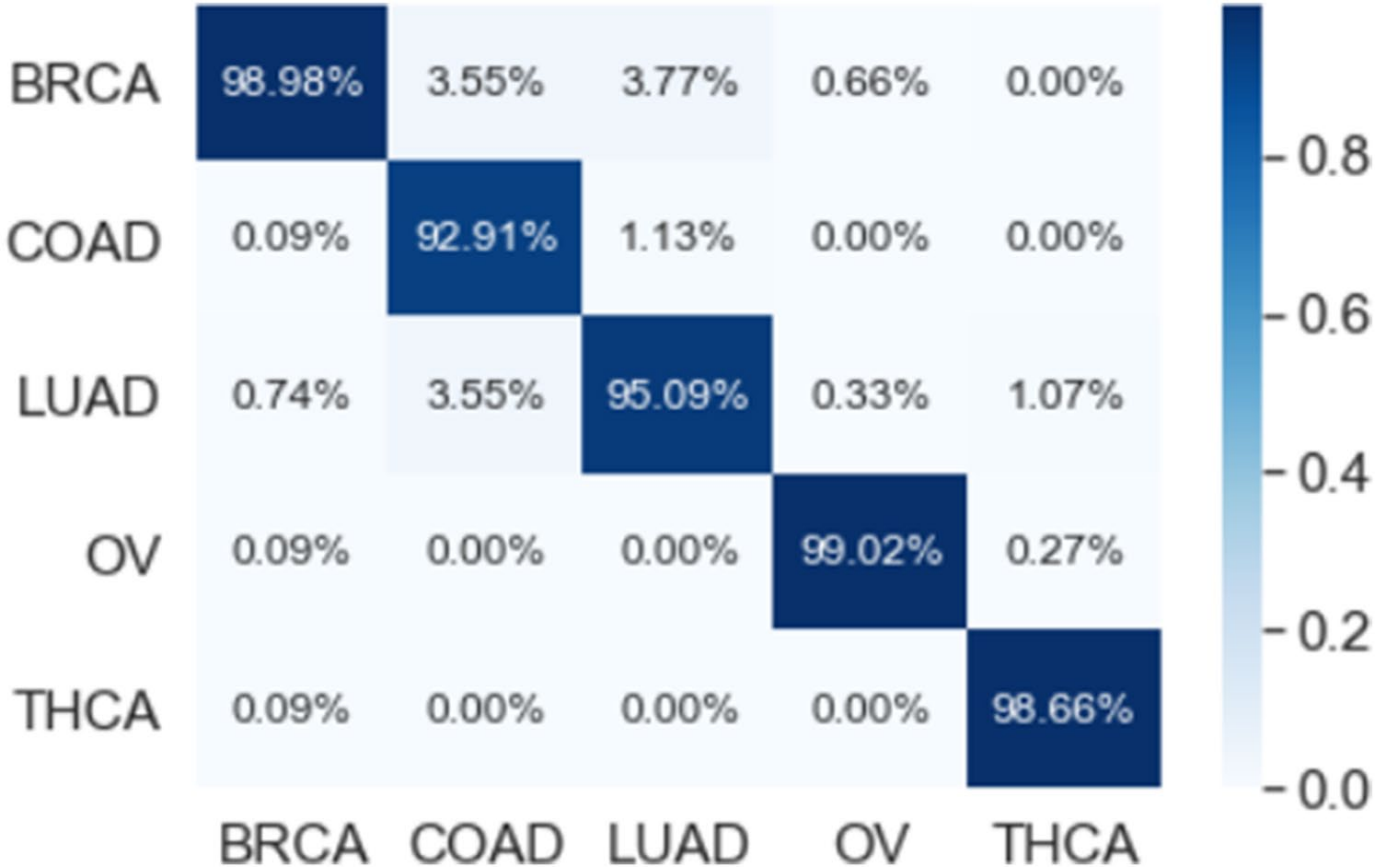
10-folds overlapped confusion matrix (CM) for all 14,899 genes.

**Figure 17:**
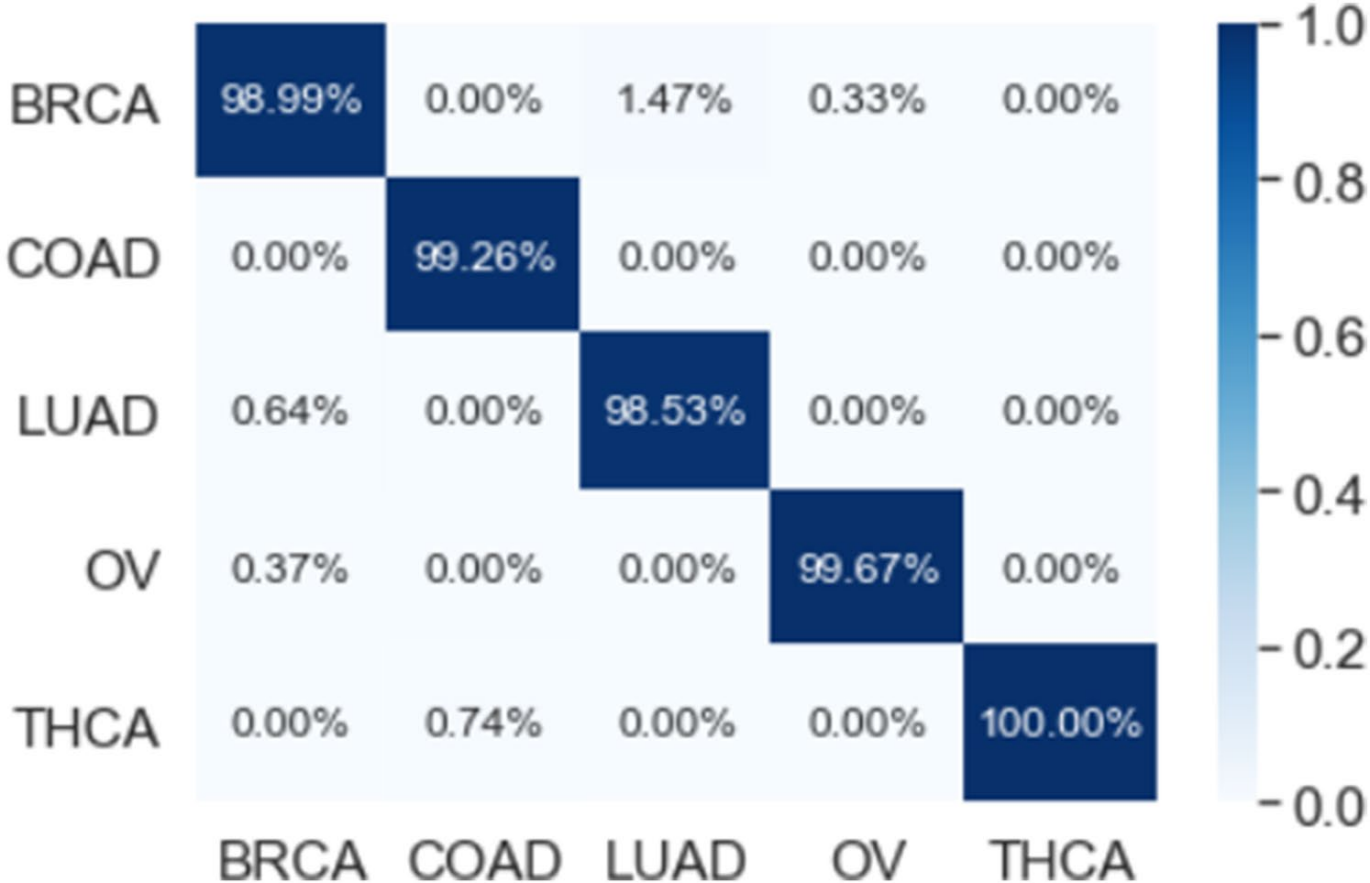
10-folds overlapped confusion matrix (CM) for the reduced 173 genes.

The multi-class classification performance of the 1D-CNN model has been evaluated for each fold, and the average classification performance of the model is calculated. The overlapped confusion matrix (CM) is shown in Figs. 16 and 17 for all and reduced lists of genes, respectively. The overlapped CM is created using the sum of the ten separated confusion matrices. Thus, it is aimed to obtain an idea about the general perforations of the model.

Although we are using RNAseq data with a high number of genes, deep learning method outperformed the machine learning methods noting that a rigorous preprocessing step including a model-based approach using LASSO regression was applied to reduce the number of genes to be less than the number of observations.

**Figure 18:**
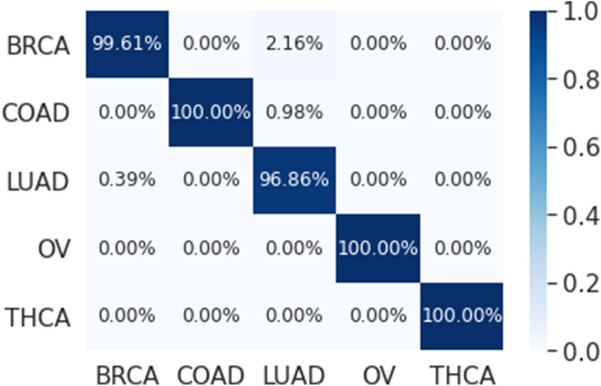
10-folds stacking ensemble deep learning model overlapped confusion matrix (CM) for all 14,899 genes.

**Figure 19:**
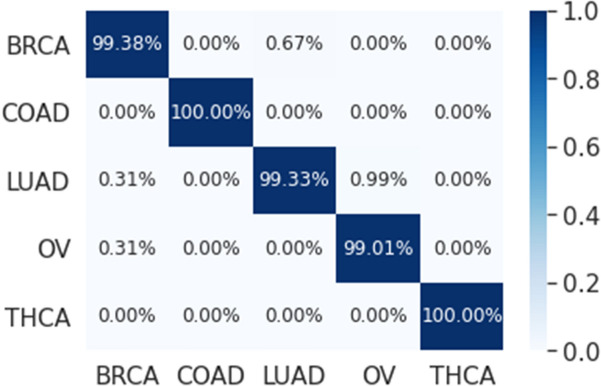
10-folds stacking ensemble deep learning model overlapped confusion matrix (CM) for the reduced 173 genes.

The results that are presented in Table 5 below show that our proposed model has a high performance when applied on the genes that are selected using LASSO (173 genes) where it achieved an average precision, recall, and F1-Score of 99.55, 99.29, and 99.42 respectively. While the classification accuracy is 99.45% which is lower compared to accuracy of the full genes. These results also showed that our proposed model outperformed the results of the single 1D-CNN model and machine learning that are presented in Tables 2 and 4. In addition, Figs. 18 and 19 which is the overlapped confusion show that our proposed model has a better classification performance compared compared to the single 1D-CNN. Overall, our proposed model performance without using LASSO as a feature selection method is comparable to the performance with LASSO.

**Table 5:** The performance of the new proposed model using early stopping regularization.

Performance measures	Folds										Overall
	1	2	3	4	5	6	7	8	9	10	
**All (14,899 genes)**											
Accuracy	99.45	99.26	99.63	99.08	99.63	99.45	99.63	99.45	99.63	99.63	99.48
Precision	99.23	99.15	99.57	98.57	99.57	99.23	99.57	99.23	99.57	99.57	99.33
Recall	98.88	98.53	99.57	98.12	99.57	99.50	99.57	98.88	99.57	99.57	99.18
F1-score	99.05	98.83	99.57	98.31	99.57	99.36	99.57	99.05	99.57	99.57	99.25
**Reduced (173 genes)**											
Accuracy	99.45	99.26	99.26	99.26	99.45	99.45	99.45	99.63	99.82	99.45	99.45
Precision	99.58	99.31	99.13	99.31	99.58	99.60	99.58	99.65	99.93	99.79	99.55
Recall	99.19	99.12	99.31	99.12	99.19	99.38	99.19	99.47	99.72	99.19	99.29
F1-score	99.38	99.22	99.22	99.22	99.38	99.49	99.38	99.56	99.82	99.49	99.42

A comparison of the methods was statistically conducted using the pairwise analysis test which produced pairwise statistical significance table of scores where the lower diagonal of the table shows p-values for the null hypothesis (distributions are the same), smaller p-value is indicative of a better model. The upper diagonal of the table presents the estimated differences in mean accuracy and kappa coefficient between the distributions. From Table 6 (under-sampling technique) we can see clearly of the fifteen pairwise comparisons of the six machine learning methods, there are nine comparisons showing statistically significant differences in terms of accuracy at the 0.05 level of significance. These differences are SVMR differed statistically to SVMP *p* = 0.003, ANN *p* =  < 0.001, and KNN *p* =  < 0.001. While SVML differed statistically to ANN *p* = 0.009, and SVMP differed statistically to ANN *p* =  < 0.001 and KNN *p* =  < 0.001. Moreover, ANN differed statistically to bagging trees *p* =  < 0.001, as well as KNN differed statistically to bagging trees *p* = 0.004.

**Table 6:** Pairwise statistical analysis test p-values and the estimated differences for the machine learning models (under-sampling technique).

	SVMR	SVML	SVMP	ANN	KNN	Bagging trees
**Accuracy**						
SVMR		0.015	− 0.015	0.138	0.038	− 0.003
SVML	1.00		− 0.030	0.123	0.022	− 0.019
SVMP	0.003	0.347		0.153	0.052	0.011
ANN	< 0.001	0.009	< 0.001		− 0.101	− 0.142
KNN	< 0.001	1.00	< 0.001	0.008		− 0.041
Bagging trees	1.00	1.00	0.250	< 0.001	0.004	
**Kappa**						
SVMR		0.024	− 0.021	0.194	0.054	− 0.005
SVML	1.00		− 0.045	0.170	0.030	− 0.029
SVMP	0.003	0.386		0.215	0.075	0.016
ANN	< 0.001	0.010	< 0.001		− 0.140	− 0.199
KNN	< 0.001	1.00	< 0.001	0.006		− 0.059
Bagging trees	1.00	1.00	0.250	< 0.001	0.004	

## Discussion

We applied a novel stacking ensemble deep learning model to classify five common cancers among women: breast, colon adenocarcinoma, lung adenocarcinoma, ovarian, and thyroid cancers. The performance of the current proposed model is compared with the single 1D-CNN and machine learning methods that are mostly used in cancer types classification. We showed that the best machine learning average results were obtained using 173 genes based on the under-sampling technique, while our proposed model has the highest performance based on the early stopping regularization. The improvement in accuracy was achieved by optimizing several parameters. We used LASSO as a feature selection technique with our proposed model to explore the integration of features selection method with a deep learning approach because features selection in deep learning is still unexplored area due to the black box nature of the deep learning methods. The results of the proposed model without using LASSO as a feature selection technique is comparable to the results with LASSO. This indicates that the 1D-CNN performs features selection through its layers. Bagging trees obtained excellent results, with a maximum accuracy of 99.2% among the machine learning models based on the under-sampling technique. In contrast, ANN showed the least accuracy of 80.7% for classifying the most common cancers among females. The SVM-P method showed performances that was close to the bagging trees method with an accuracy of 98.9% when we used the under-sampling technique. Overall, our results showed that SVM-R, SVM-L, SVM-P, ANN, KNN, and bagging trees were improved in performance if under-sampling is applied compared to over-sampling. We conclude that our proposed model is the best methods for the test dataset in this study. However, bagging trees is the best model among the machine learning models.

Overall, our proposed model surpassed the single 1D-CNN and the machine learning methods in the classification of common cancers among women. These findings are different from those reported in other studies^11,18,19^. These differences can be explained by variations in the type of cancers studied and the methods used for feature/gene selection. A study by Yang and Naiman^14^ introduced and validated a gene selection approach using machine learning methods but did not assess the performance of the machines. Our findings demonstrated that, our proposed model can achieve a higher performance on cancer tumor classification using gene expression data. Both deep and machine learning methods and a combination of both can assist in predicting or detecting cancer susceptibility in the early stages and therefore, aid in designing early treatment strategies, and in turn increase survival of the high-risk women.

Because of the large number of genes in the gene expression data, we used LASSO regression as a rigorous feature selection method that reduced the dimensionality of the data sets^24,68^. This process enabled us to retain the most important features (genes) for classification and prediction. In order to avoid over-fitting and the bias in the skewed class distribution we used over and under-sampling imbalance handling techniques, which improve the machine learning performance. In general, our results show that under-sampling technique improved the methods performance, and this is confirmed in previous studies^64,65,69^.

There were statistically significant differences (*p* < 0.05) between the machine learning methods, which demonstrates that the performance of the machines on cancer classification is not the same. However, deep learning methods outperformed the machine learning methods in cancer classification, which is similar to a previous study^23^. Overall, the accuracy of our proposed model on the full features and on the features that are selected using LASSO are 99.48% and 99.45, respectively, which are 5.05% and 5.02% higher than accuracy obtained by^23^ which is 94.43%. We note that Tabares-Soto et al.^24^ used microarray gene expression data, focusing on 11 type of cancers for both males and females, compared to RNASeq data used in this study to classify five common cancers among females. This study also did not consider class imbalance handling methods as applied in the current study and had 12-times lower sample size (n = 174) than in our study (n = 2166). With larger sample size, more samples are available to train the models. These issues were, therefore, likely to affect the reliability of findings and potentially affecting the performance of the methods. Our study was limited to the gene expression profiles from RNASeq data. However, Lee and co-workers^22^ used several features such as mutation profiles and mutations rates. They evaluated different machine learning and feature selection methods using RNASeq data from 31 cancer types. The highest accuracy they obtained was 84%. Thereafter, they reduced the number of cancers to the six most common types and obtained an accuracy of 94%, which is low compared to our proposed deep learning model.

Our proposed model has a very high achievement in classifying the five common cancers among women and that may potentially improve the multi-class identification^19^. In addition, this study is first of its kind to classify cancer tumors using RNAseq data. However, multi-class cancer classification using gene expression is not a substitute to the traditional diagnosis^19^, but advances in classification algorithms or methods may provide a more accurate and biologically meaningful classifications and inform future studies. Moreover, a more pressing classification problem may be that of discriminating between cancer sub-types within the same type than between cancer types. However, we postulate that the methods covered in this paper are directly applicable to this problem.

## Conclusion

In this work, we proposed a stacking ensemble deep learning model as a multi-class classifier to classify five most common cancers among women, that is, breast, colon adenocarcinoma, lung adenocarcinoma, ovarian, and thyroid cancer, using RNASeq gene expression datasets for each cancer tumor. Tumor classification using RNASeq data is more accurate and available compared to microarray data. We used LASSO as a feature selection method and compared the performance of our proposed method with a stand alone deep learning and machine learning methods. We conclude that our proposed model achieved the highest performance compared to the single 1D-CNN and the machine learning methods. Our proposed model is, therefore, capable of correctly classifying all the observed positive cancer cases. The proposed model can help improve the detection and diagnosis of cancer susceptibility among women in the early stages, inform decision on early intervention, and hence improve survival. Future research should consider the potential effects of using many feature types such as methylations and mutations, to be integrated with RNASeq data. Future work will also consider improvements on the stacking ensemble problem including statistical properties to improve inference.

## Supplementary Information

Supplementary Information 1.

Supplementary Information 2.

## Data Availability

The datasets are publicly available on The Cancer Genome Atlas (TCGA) repository.
