# Supplementary material for: A stacking ensemble deep learning approach to cancer type classification based on TCGA data
Source: Sci Rep. 2021 Aug 2;11:15626. doi: 10.1038/s41598-021-95128-x (PMC8329290; doi:10.1038/s41598-021-95128-x)
Supplement: Supplementary file 1 — Supplementary Information 1. [file 41598_2021_95128_MOESM1_ESM.pdf]

A Stacking Ensemble Deep Learning Approach to Cancer Type Classification Based on TCGA Data

Mohanad Mohammed[1*], Henry Mwambi[1], Innocent B. Mboya[1,4], Murtada K. Elbashir[5,6], Bernard Omolo[1,2,3]

[1]School of Mathematics, Statistics and Computer Science, University of KwaZulu-Natal, Pietermaritzburg, Private Bag X01, Scottsville 3209, South Africa.

[2]Division of Mathematics & Computer Science, University of South Carolina-Upstate, 800 University Way, Spartanburg, USA.

[3]School of Public Health, Faculty of Health Sciences, University of Witwatersrand, Johannesburg, South Africa.

[4]Department of Epidemiology and Biostatistics, Kilimanjaro Christian Medical University College (KCMUCo), P. O. Box 2240, Moshi-Tanzania.

[5]College of Computer and Information Sciences, Jouf University, Sakaka 72441, Saudi Arabia.

[6]Faculty of Mathematical and Computer Sciences, University of Gezira, Wad Madani 11123, Sudan.

* Corresponding Author, mohanadadam32@gmail.com

The 173 significant genes that were returned using LASSO with 10-folds cross-validation.

	baseMean	log2FoldChange	lfcSE	stat	pvalue	padj
**ACSL4**	3370.81	0.86896	0.0476	18.2555	1.87E-74	5.87E-74
**ADAM28**	528.011	-0.6334	0.07523	-8.4205	3.75E-17	5.89E-17
**ADAM8**	1711.32	-1.6217	0.06691	-24.236	9.37E-130	4.72E-129
**AFF1**	6892.33	1.91525	0.03596	53.2632	0	0
**ALPK3**	806.889	1.03086	0.06688	15.4126	1.35E-53	3.41E-53
**ANKS4B**	185.576	-4.4648	0.3449	-12.945	2.50E-38	5.34E-38
**ANXA6**	9806.23	-0.8436	0.04146	-20.347	4.90E-92	1.80E-91
**AQP3**	5437.67	-4.0455	0.10407	-38.875	0	0
**AQP4**	906.546	4.21352	0.14895	28.2886	4.77E-176	3.38E-175
**ATF1**	1520.23	0.63084	0.0224	28.1681	1.44E-174	1.01E-173
**ATP2B4**	11058.4	-0.1707	0.04502	-3.791	0.00015007	0.00018147
**ATP6V0C**	11556.3	-0.2019	0.02699	-7.4806	7.40E-14	1.10E-13
**BDH2**	2407.05	2.14255	0.04332	49.4594	0	0
**C11orf41**	550.413	-2.9532	0.09899	-29.835	1.39E-195	1.13E-194
**C18orf45**	1239.63	-2.9454	0.03854	-76.428	0	0
**C1orf186**	549.158	-1.0929	0.12461	-8.7706	1.78E-18	2.86E-18
**C1orf190**	113.125	3.35189	0.06494	51.6182	0	0
**C2orf89**	603.527	2.286	0.08833	25.8802	1.11E-147	6.40E-147
**C5orf53**	849.576	1.31961	0.03811	34.6256	1.04E-262	1.29E-261
**C6orf138**	223.422	4.66633	0.09545	48.8861	0	0
**C6orf222**	123.793	-1.3166	0.22378	-5.8834	4.02E-09	5.43E-09
**C9orf167**	1440.81	2.15425	0.04608	46.753	0	0
**C9orf91**	1988.46	-1.8341	0.03453	-53.118	0	0
**CCDC109A**	1907.7	-0.1384	0.03195	-4.331	1.48E-05	1.84E-05
**CCL13**	213.252	1.6253	0.12751	12.7464	3.27E-37	6.87E-37
**CCNJL**	633.306	1.36706	0.06965	19.6283	8.87E-86	3.08E-85
**CDH26**	72.9319	-0.8001	0.10072	-7.9431	1.97E-15	3.00E-15
**CDX1**	673.477	-1.5722	0.0964	-16.309	8.51E-60	2.30E-59
**CEACAM6**	28629.9	-3.6857	0.14666	-25.131	2.27E-139	1.23E-138
**CFL2**	1388.26	1.19518	0.0398	30.0259	4.51E-198	3.73E-197
**CHRAC1**	2544.38	0.15682	0.03415	4.59235	4.38E-06	5.52E-06
**CHSY1**	3327.68	-1.3737	0.0392	-35.046	4.43E-269	5.71E-268
**CLDN3**	11251.4	1.37722	0.06213	22.1666	7.22E-109	3.09E-108
**CPNE8**	456.31	0.57156	0.05593	10.219	1.63E-24	2.90E-24
**CTSB**	111838	3.01424	0.04021	74.9717	0	0
**CTSK**	7158.98	-2.5965	0.07718	-33.639	4.45E-248	5.05E-247
**CTU1**	275.536	0.30631	0.04339	7.05937	1.67E-12	2.42E-12
**CXCL17**	3781.26	-0.7251	0.14759	-4.9128	8.98E-07	1.15E-06
**CYP2C18**	63.4312	-4.0536	0.26513	-15.289	9.07E-53	2.27E-52
**DCBLD2**	4066.26	2.03321	0.05135	39.5971	0	0

**DFNA5**	605.619	1.6637	0.0525	31.6899	2.14E-220	2.07E-219
**DIRAS1**	474.766	-2.5309	0.09457	-26.761	9.16E-158	5.69E-157
**DOCK3**	714.113	4.56055	0.09507	47.9697	0	0
**DPY19L1**	2490.91	0.73826	0.04509	16.3721	3.03E-60	8.24E-60
**DVL3**	6057.44	-0.4287	0.0273	-15.702	1.46E-55	3.79E-55
**EFS**	1994.72	-1.8672	0.0705	-26.485	1.42E-154	8.63E-154
**EMX2**	1161.03	-1.1471	0.12748	-8.998	2.30E-19	3.75E-19
**ENO1**	57304.3	-0.3479	0.04262	-8.1623	3.29E-16	5.08E-16
**EPHA2**	2478.12	2.20884	0.05574	39.6304	0	0
**ERO1L**	3225.93	-0.3541	0.04669	-7.5841	3.35E-14	5.00E-14
**ERRFI1**	7027.42	0.95015	0.06015	15.7957	3.33E-56	8.70E-56
**F2RL2**	946.889	-4.1363	0.09586	-43.152	0	0
**FAM115C**	640.22	-1.7596	0.05678	-30.989	7.61E-211	6.82E-210
**FAM126A**	1043.82	-0.6537	0.05516	-11.849	2.17E-32	4.30E-32
**FAM49A**	457.997	2.93101	0.05577	52.5511	0	0
**FOXE1**	2538.15	11.0891	0.20785	53.3515	0	0
**FUT4**	749.106	-0.3538	0.04268	-8.2898	1.13E-16	1.77E-16
**FYN**	3259.73	2.47148	0.05261	46.9818	0	0
**FZD5**	1539.99	2.10413	0.05529	38.0591	0	0
**GALNT12**	1395.26	3.90386	0.07389	52.8336	0	0
**GAS2L1**	1933.06	-0.8869	0.03449	-25.712	8.53E-146	4.82E-145
**GGCX**	2233.96	-0.2142	0.03163	-6.77	1.29E-11	1.83E-11
**GJD3**	218.919	0.76789	0.06063	12.665	9.24E-37	1.93E-36
**GMDS**	1379.91	-1.0039	0.04837	-20.755	1.11E-95	4.22E-95
**GPR35**	419.506	-0.7246	0.06243	-11.608	3.77E-31	7.36E-31
**GREM1**	2587.79	-2.7903	0.10787	-25.868	1.52E-147	8.73E-147
**GTF3C3**	1987.34	-0.3592	0.02385	-15.062	2.89E-51	7.13E-51
**GULP1**	640.82	0.34179	0.07153	4.77814	1.77E-06	2.25E-06
**HMG20B**	5489.95	-0.8186	0.02685	-30.484	4.31E-204	3.71E-203
**HMGB3**	5577.2	-1.6441	0.06003	-27.388	3.86E-165	2.52E-164
**HNF1A**	245.61	-1.1339	0.07841	-14.461	2.14E-47	5.07E-47
**HNF4G**	277.795	-6.2544	0.13349	-46.852	0	0
**HOXA13**	77.3845	-2.3855	0.20455	-11.662	1.99E-31	3.89E-31
**HOXC8**	167.777	-5.4351	0.0949	-57.275	0	0
**HOXC9**	218.878	-5.1496	0.09837	-52.347	0	0
**IER5L**	2059.74	0.28554	0.05433	5.25538	1.48E-07	1.93E-07
**IGF2BP1**	142.698	-2.2957	0.18221	-12.6	2.12E-36	4.41E-36
**IL22RA1**	266.713	2.25289	0.09592	23.4876	5.46E-122	2.56E-121
**IPPK**	1053.05	-1.0976	0.03032	-36.207	4.81E-287	6.90E-286
**IQCA1**	828.264	3.48015	0.08491	40.9859	0	0
**IRAK3**	760.228	-0.8446	0.07413	-11.394	4.46E-30	8.57E-30
**IRX5**	1464.78	-7.411	0.08945	-82.853	0	0

**ISCU**	5100.68	1.90679	0.02875	66.3119	0	0
**ITGB3**	1998.06	4.17437	0.08147	51.2376	0	0
**KCNJ15**	2194.64	6.55889	0.08335	78.6886	0	0
**KLF3**	4155.05	0.39248	0.02462	15.9413	3.27E-57	8.63E-57
**KLHL14**	1062.48	5.21229	0.10642	48.9801	0	0
**LILRB3**	177.152	-0.478	0.06915	-6.9133	4.74E-12	6.78E-12
**LMO7**	6171.05	2.30817	0.04991	46.2485	0	0
**LOC84740**	930.367	-4.7457	0.15509	-30.6	1.21E-205	1.05E-204
**LPCAT1**	8849.05	-1.1973	0.04956	-24.157	6.28E-129	3.14E-128
**MAFK**	2562.79	0.24663	0.03922	6.28828	3.21E-10	4.44E-10
**MAPKAPK3**	3705.76	1.74455	0.03236	53.9119	0	0
**MEIS1**	2136.62	-1.7935	0.05538	-32.387	4.14E-230	4.25E-229
**MUC4**	1639.02	-0.5678	0.15128	-3.7534	0.000174446	0.000210365
**NBPF10**	2075.07	-1.1899	0.05655	-21.041	2.74E-98	1.07E-97
**NIN**	2548.78	-0.8453	0.03187	-26.528	4.66E-155	2.83E-154
**NUDT16P1**	458.128	1.45315	0.06586	22.0633	7.11E-108	3.02E-107
**NYNRIN**	2599.49	1.2127	0.05817	20.8465	1.64E-96	6.29E-96
**OGFRL1**	1005.35	-1.5405	0.05503	-27.992	2.02E-172	1.39E-171
**OSBPL3**	1900.07	1.40025	0.0519	26.9776	2.70E-160	1.71E-159
**PABPC3**	1407.18	-0.9747	0.04857	-20.067	1.43E-89	5.15E-89
**PADI2**	2795.44	-4.4786	0.09602	-46.642	0	0
**PAPLN**	1439.99	2.47032	0.0647	38.1831	0	0
**PCDP1**	155.199	1.20581	0.13261	9.093	9.63E-20	1.58E-19
**PIK3R1**	5504.79	-0.687	0.05038	-13.637	2.43E-42	5.44E-42
**PITPNM1**	3075.04	1.17408	0.04443	26.4284	6.46E-154	3.89E-153
**PNRC1**	6180.57	0.64155	0.03687	17.4	8.25E-68	2.43E-67
**POF1B**	1116.43	-3.0652	0.12466	-24.588	1.70E-133	8.85E-133
**POSTN**	36945.3	-3.1331	0.09535	-32.86	8.24E-237	8.75E-236
**PPP2R4**	10291.5	-0.539	0.02472	-21.806	2.03E-105	8.42E-105
**PPP4R1**	4565.9	0.19383	0.0274	7.07291	1.52E-12	2.19E-12
**PRIMA1**	426.151	-1.4497	0.10554	-13.737	6.11E-43	1.38E-42
**PROX1**	94.9841	1.67423	0.10665	15.6988	1.54E-55	3.99E-55
**PRR7**	356.667	-0.7432	0.06295	-11.807	3.59E-32	7.08E-32
**PRSS3**	347.489	1.20214	0.168	7.15547	8.34E-13	1.21E-12
**PSME4**	5848.39	-0.896	0.03328	-26.926	1.08E-159	6.81E-159
**PTGS1**	3620.61	-1.3029	0.05834	-22.333	1.77E-110	7.69E-110
**RAB11FIP5**	2452.82	-0.6883	0.03307	-20.812	3.35E-96	1.28E-95
**RAB3IP**	1843.85	-0.4525	0.04814	-9.399	5.51E-21	9.25E-21
**RBM14**	3005.43	-0.1571	0.01623	-9.6794	3.69E-22	6.32E-22
**RBMXL1**	1757.42	-0.1196	0.02259	-5.2947	1.19E-07	1.56E-07
**REP15**	67.4638	0.57378	0.06699	8.56567	1.07E-17	1.71E-17
**RHOF**	1401.89	1.75816	0.07418	23.7008	3.54E-124	1.69E-123

**RHOU**	3104.79	2.1605	0.06005	35.9766	1.94E-283	2.71E-282
**ROS1**	587.327	-1.0717	0.22531	-4.7565	1.97E-06	2.51E-06
**RTN4RL1**	820.116	-4.1761	0.09025	-46.273	0	0
**SEL1L3**	5779.97	2.04867	0.06482	31.6048	3.17E-219	3.04E-218
**SFTA2**	860.678	4.31509	0.18356	23.5073	3.43E-122	1.61E-121
**SFTPA2**	26597.7	4.01867	0.15964	25.1727	7.98E-140	4.34E-139
**SFTPB**	58184.4	10.7305	0.18489	58.0387	0	0
**SGPP2**	505.305	1.69707	0.0884	19.198	3.85E-82	1.29E-81
**SH3TC2**	137.14	-0.3047	0.08531	-3.5711	0.000355476	0.000424991
**SHOX2**	137.388	-2.6585	0.08671	-30.659	2.01E-206	1.75E-205
**SLC16A3**	4475.32	0.2324	0.06165	3.76938	0.000163654	0.000197543
**SLC5A6**	3718.44	-3.0248	0.0499	-60.621	0	0
**SNRPN**	7199.12	2.20516	0.05457	40.4128	0	0
**SNTB1**	2940.19	2.87747	0.0581	49.5222	0	0
**SOX17**	1447.5	0.32306	0.05814	5.55664	2.75E-08	3.64E-08
**SOX2**	328.115	-5.2739	0.18675	-28.241	1.86E-175	1.31E-174
**SPRR3**	50.4587	-3.0802	0.2934	-10.498	8.83E-26	1.59E-25
**SRL**	342.905	5.44947	0.07675	71.0073	0	0
**STAMBPL1**	350.138	-0.6987	0.05128	-13.624	2.87E-42	6.43E-42
**STARD3NL**	1970.17	0.60911	0.02799	21.7602	5.53E-105	2.29E-104
**STK17A**	1733.47	-0.4008	0.041	-9.7763	1.42E-22	2.46E-22
**STK33**	306.893	4.99973	0.10542	47.4282	0	0
**TBX4**	73.5169	-3.1685	0.15579	-20.339	5.82E-92	2.13E-91
**TBX5**	141.449	-2.4714	0.10744	-23.003	4.34E-117	1.97E-116
**TFAP2A**	3570.45	-7.265	0.07658	-94.868	0	0
**TFPI**	2180.11	-0.8757	0.07368	-11.886	1.41E-32	2.79E-32
**TG**	122733	14.6101	0.09508	153.656	0	0
**TMEM125**	1976.24	0.60393	0.0452	13.3617	1.01E-40	2.23E-40
**TMEM189.UBE2V1**	106.474	-0.9133	0.18485	-4.9407	7.78E-07	1.00E-06
**TMPRSS4**	3172.42	3.24068	0.13923	23.2751	7.92E-120	3.68E-119
**TMUB1**	2457.84	0.38033	0.02643	14.3878	6.17E-47	1.45E-46
**TNFSF10**	8460.82	-2.8268	0.07024	-40.246	0	0
**TP53I3**	1034.62	0.86222	0.04468	19.2996	5.41E-83	1.83E-82
**TRPS1**	14055.4	-4.6335	0.05918	-78.295	0	0
**TSHR**	5366.57	10.606	0.10119	104.809	0	0
**TSHZ2**	342.181	-1.2576	0.07492	-16.785	3.12E-63	8.76E-63
**TSPAN3**	15911.8	1.43967	0.03965	36.3094	1.15E-288	1.67E-287
**TXNRD1**	6856.62	-0.6134	0.04865	-12.61	1.87E-36	3.89E-36
**UAP1**	4251.85	-1.5417	0.03362	-45.86	0	0
**UBAP1**	3893.18	-0.0895	0.02371	-3.7774	0.000158448	0.000191353
**VANGL1**	2792.57	-1.1929	0.03663	-32.563	1.37E-232	1.43E-231
**VSTM2L**	1170.07	1.22453	0.11733	10.4363	1.69E-25	3.04E-25

**WT1**	1797.4	-4.7802	0.1424	-33.569	4.83E-247	5.48E-246
**XAGE1D**	1129.49	-4.7626	0.30729	-15.499	3.53E-54	9.00E-54
**ZDHHC7**	3272.22	-0.1507	0.02559	-5.8885	3.90E-09	5.27E-09
**ZNF280B**	198.397	2.01472	0.0844	23.8724	5.93E-126	2.89E-125
**ZNF628**	562.854	0.27863	0.0287	9.70931	2.75E-22	4.73E-22
**ZNF771**	256.77	-0.2152	0.04531	-4.7499	2.04E-06	2.59E-06
**ZNF90**	1110.07	1.3678	0.06389	21.4085	1.11E-101	4.47E-101
