# Supplementary material for: A stacking ensemble deep learning approach to cancer type classification based on TCGA data
Source: Sci Rep. 2021 Aug 2;11:15626. doi: 10.1038/s41598-021-95128-x (PMC8329290; doi:10.1038/s41598-021-95128-x)
Supplement: Supplementary file 2 — Supplementary Information 2. [file 41598_2021_95128_MOESM2_ESM.pdf]

A Stacking Ensemble Deep Learning Approach to Cancer Type Classification Based on TCGA Data

Mohanad Mohammed[1*], Henry Mwambi[1], Innocent B. Mboya[1,4], Murtada K. Elbashir[5,6], Bernard Omolo[1,2,3]

[1]School of Mathematics, Statistics and Computer Science, University of KwaZulu-Natal, Pietermaritzburg, Private Bag X01, Scottsville 3209, South Africa.

[2]Division of Mathematics & Computer Science, University of South Carolina-Upstate, 800 University Way, Spartanburg, USA.

[3]School of Public Health, Faculty of Health Sciences, University of Witwatersrand, Johannesburg, South Africa.

[4]Department of Epidemiology and Biostatistics, Kilimanjaro Christian Medical University College (KCMUCo), P. O. Box 2240, Moshi-Tanzania.

[5]College of Computer and Information Sciences, Jouf University, Sakaka 72441, Saudi Arabia.

[6]Faculty of Mathematical and Computer Sciences, University of Gezira, Wad Madani 11123, Sudan.

* Corresponding Author, mohanadadam32@gmail.com

Results

The overall predictive performance of the machine learning methods based on the oversampling

Table 1. The overall predictive performance of the machine learning methods based on the oversampling

Methods	Performance Measures					
	**ACC (95% CI)**	**Kappa (95% CI)**	**F1-Score**	**Precision**	**Sensitivity**	**AUC**
**SVM-R**	93.1 (90.8, 94.9)	89.6 (86.8, 92.5)	97.6	99.4	95.9	97.2
**SVM-L**	82.4 (79.3, 85.3)	71.9 (67.5, 76.3)	88.1	100.0	78.7	92.0
**SVM-P**	84.0 (80.9, 86.7)	75.8 (71.9, 79.6)	94.7	100.0	89.9	90.9
**ANN**	86.3 (83.4, 88.8)	80.4 (76.8, 84.1)	92.1	86.9	97.9	89.7
**kNN**	92.0 (89.6, 94.0)	88.4 (85.4, 91.3)	96.0	93.6	98.4	96.3
**Bagging**	98.0 (96.6, 98.9)	97.0 (95.4, 98.6)	98.1	100	96.2	99.4

Note: SVM-R, Support Vector Machine with Radial-basis function (RBF) kernel; SVM-L, Support Vector Machine with Linear Kernel; SVM-P, Support Vector Machine with Polynomial Kernel; ANN, Artificial Neural Networks; kNN, K-nearest Neighbors; ACC, Accuracy; CI, Confidence Interval; Kappa, Kappa Statistics; AUC, Area Under the Curve.

Predictive performance of the machine learning methods per cancer type based on the oversampling

Table 2. Predictive performance of the machine learning methods per-class statistics based on the oversampling

Performance Measures	Methods						
	**Class**	**SVM-R**	**SVM-L**	**SVM-P**	**ANN**	**kNN**	**Bagging**
**Accuracy**	BRCA	97.5	86.1	94.2	92.5	96.0	98.0
	COAD	93.1	97.5	94.9	90.9	93.7	95.8
	LUAD	97.9	82.2	89.8	80.1	86.0	97.1
	OV	82.7	52.3	50.6	93.2	98.0	97.1
	THCA	96.0	99.6	99.6	98.9	99.1	100.0
**Sensitivity**	BRCA	99.4	100.0	100.0	86.9	93.6	100.0
	COAD	86.1	97.2	100.0	88.9	94.4	91.7
	LUAD	100.0	66.3	80.2	61.6	72.1	94.2
	OV	66.7	4.6	01.1	90.8	96.6	94.3
	THCA	91.9	99.1	99.1	99.1	98.2	100.0
**Specificity**	BRCA	95.6	72.2	88.4	98.1	98.4	95.9
	COAD	100.0	97.7	89.7	93.0	93.0	100.0
	LUAD	95.7	98.0	99.3	98.6	99.8	100.0
	OV	98.8	100.8	100.0	95.6	99.5	100.0
	THCA	100.0	100.0	100.0	98.7	100.0	100.0
**F1-Score**	BRCA	97.6	88.1	94.7	92.1	96.0	98.1
	COAD	92.5	82.4	53.3	57.7	60.2	95.7
	LUAD	87.8	74.0	86.8	72.1	83.2	97.0
	OV	76.3	08.8	02.3	82.7	96.6	97.0
	THCA	95.8	99.6	99.6	96.5	99.1	100.0
**Precision**	BRCA	95.9	78.7	89.9	98.0	98.4	96.2
	COAD	100.0	71.4	36.4	42.7	44.2	100.0
	LUAD	78.2	83.8	94.5	86.9	98.4	100.0
	OV	89.2	100.1	100.0	76.0	96.6	100.0
	THCA	100.0	100.0	100.0	94.0	100.0	100.0

Note: SVM-R, Support Vector Machine with Radial-basis function (RBF) kernel; SVM-L, Support Vector Machine with Linear Kernel; SVM-P, Support Vector Machine with Polynomial Kernel; ANN, Artificial Neural Networks; kNN, K-nearest Neighbors.

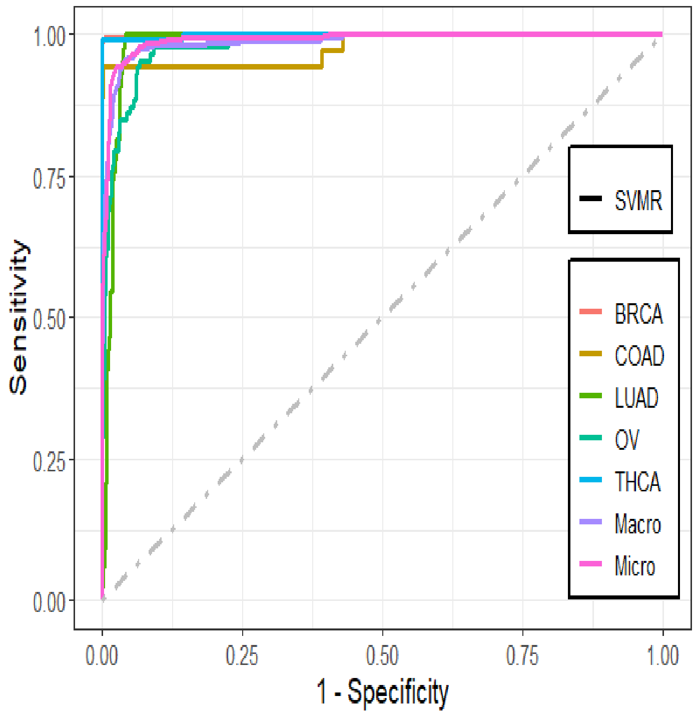

Figure 1. Multi-class ROC curves visualization for the SVMR model based on over sampling technique.

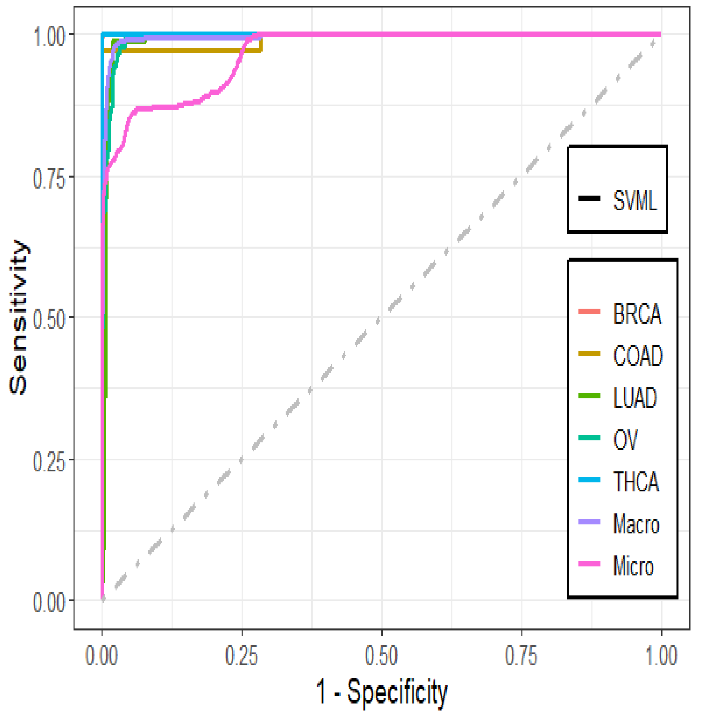

Figure 2. Multi-class ROC curves visualization for the SVML model based on over sampling technique.

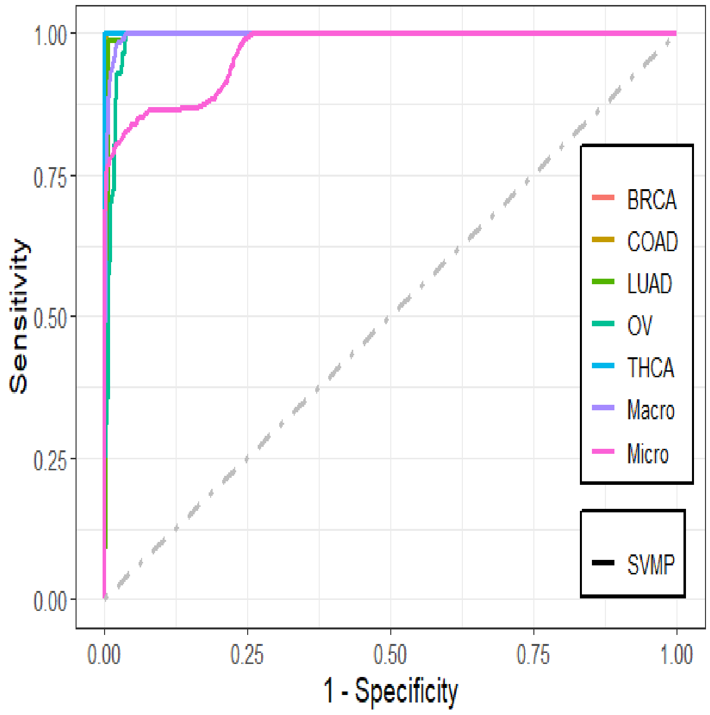

Figure 3. Multi-class ROC curves visualization for the SVMP model based on over sampling technique.

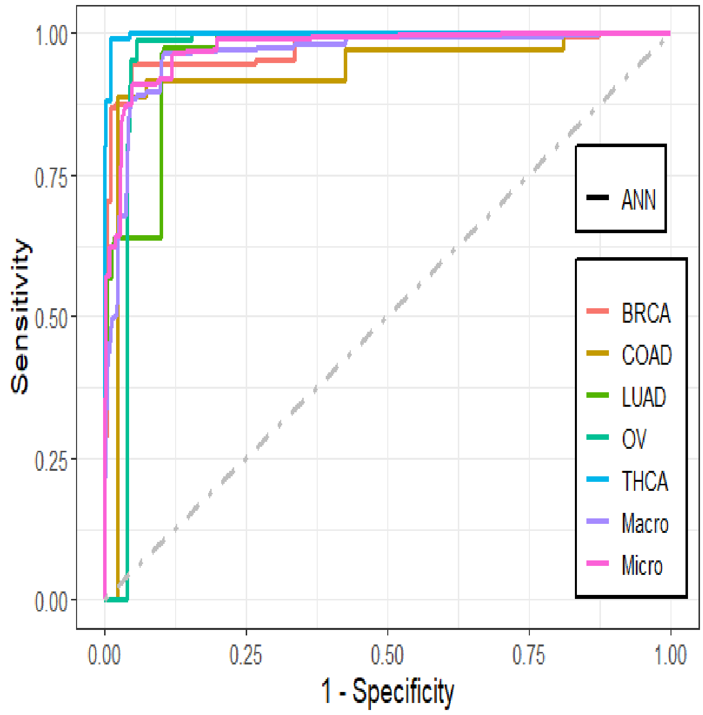

Figure 4. Multi-class ROC curves visualization for the ANN model based on over sampling technique.

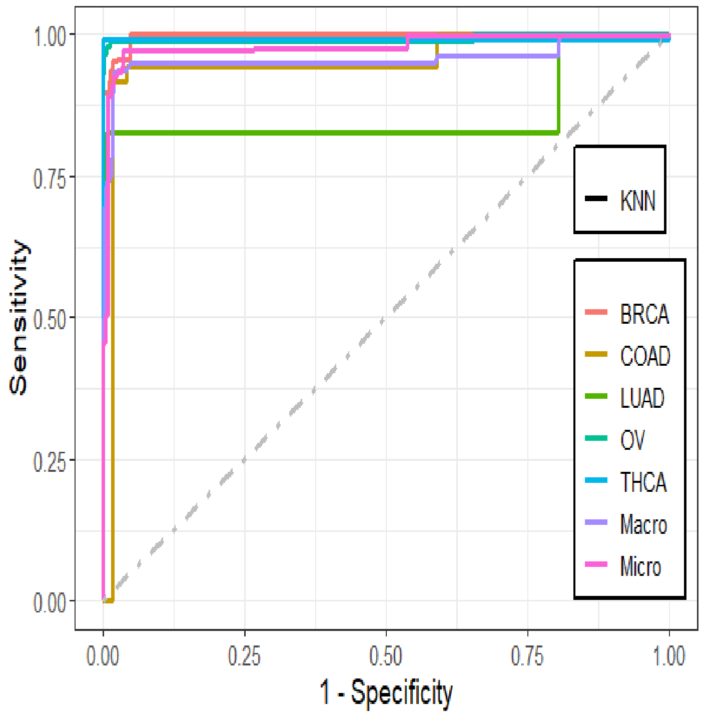

Figure 5. Multi-class ROC curves visualization for the kNN model based on over sampling technique**.**

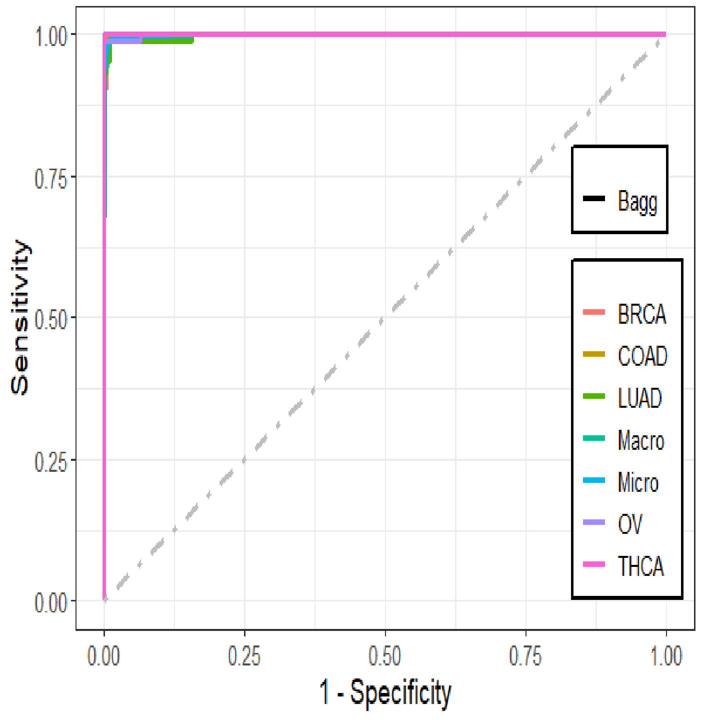

Figure 6. Multi-class ROC curves visualization for the bagging model based on over sampling technique**.**

Statistical Significance Test

Table **3**. Pairwise statistical analysis test p-values and the estimated differences for the models (over sampling technique)

			Accuracy			
	**SVMR**	**SVML**	**SVMP**	**ANN**	**KNN**	**Bagging**
**SVMR**		0.120	0.109	0.090	-0.008	-0.054
**SVML**	0.001		-0.011	-0.030	-0.128	-0.174
**SVMP**	0.001	1.00		-0.019	-0.117	-0.163
**ANN**	0.002	1.00	1.00		-0.098	-0.144
**KNN**	1.00	<0.001	<0.001	0.004		-0.045
**Bagging**	0.077	<0.001	<0.001	<0.001	<0.001	
			**Kappa**			
	**SVMR**	**SVML**	**SVMP**	**ANN**	**KNN**	**Bagging**
**SVMR**		0.193	0.162	0.135	-0.013	-0.079
**SVML**	<0.001		-0.030	-0.057	-0.206	-0.272
**SVMP**	<0.001	1.00		-0.027	-0.175	-0.241
**ANN**	0.002	1.00	1.00		-0.148	-0.214
**KNN**	1.00	<0.001	<0.001	0.004		-0.066
**Bagging**	0.076	<0.001	<0.001	<0.001	<0.001	

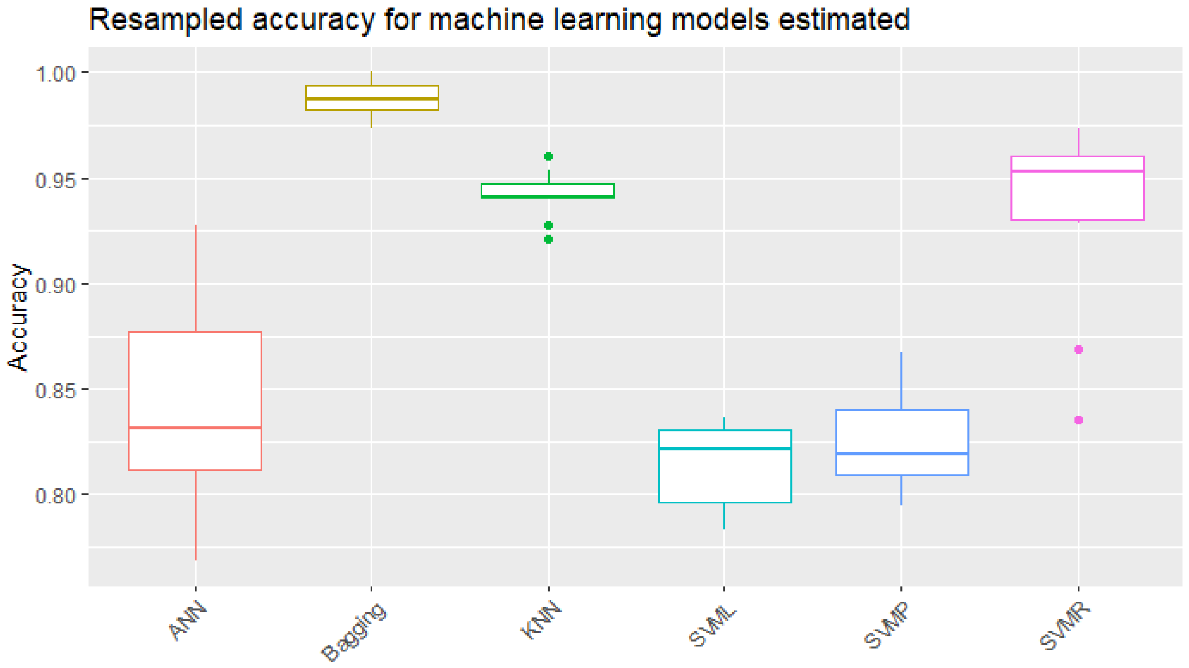

Figure 20. Compares both the mean estimated accuracy and kappa statistic as well as the 95% confidence interval for the methods based on the over sampling technique**.**

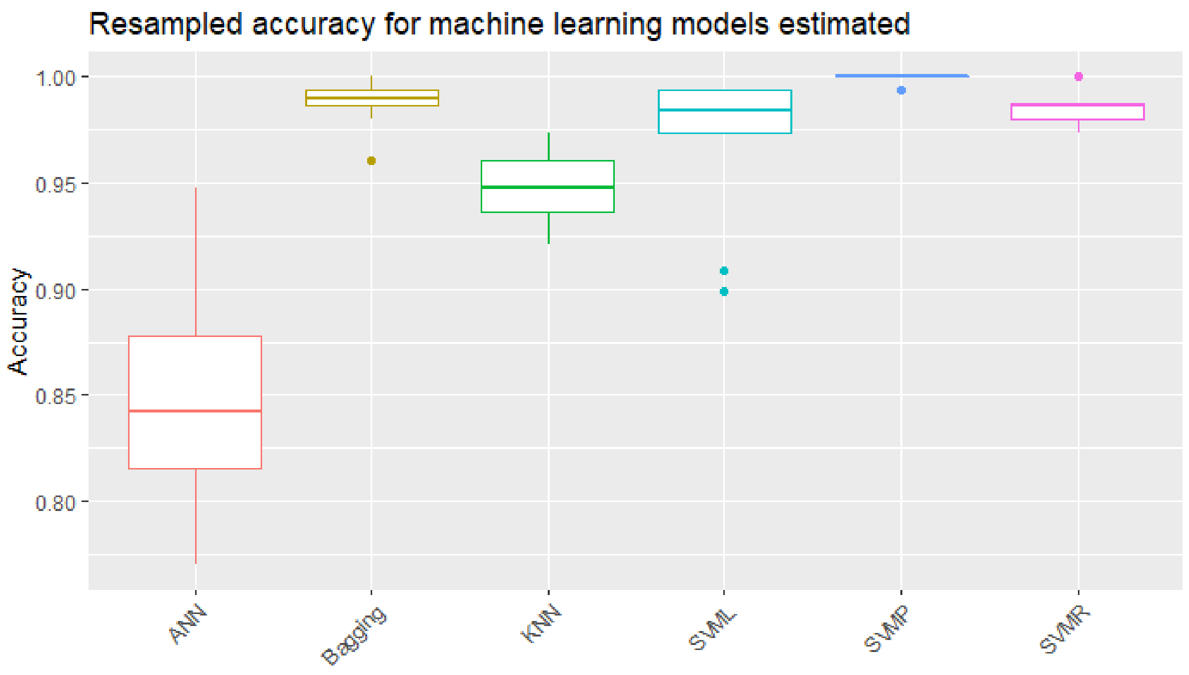

Figure 21. Compares both the mean estimated accuracy and kappa statistic as well as the 95% confidence interval for the methods based on the under sampling technique**.**
